# The HDAC/HSP90 Inhibitor G570 Attenuated Blue Light-Induced Cell Migration in RPE Cells and Neovascularization in Mice through Decreased VEGF Production

**DOI:** 10.3390/molecules26144359

**Published:** 2021-07-19

**Authors:** Tai-Ju Hsu, Kunal Nepali, Chi-Hao Tsai, Zuha Imtiyaz, Fan-Li Lin, George Hsiao, Mei-Jung Lai, Yu-Wen Cheng

**Affiliations:** 1 School of Pharmacy, College of Pharmacy, Taipei Medical University, Taipei 100301, Taiwan; nick010519@tmu.edu.tw (T.-J.H.); nepali@tmu.edu.tw (K.N.); d01447001@ntu.edu.tw (C.-H.T.); zuhaimtiyaz@tmu.edu.tw (Z.I.); 2 Department of Ophthalmology, School of Medicine, The University of North Carolina at Chapel Hill, Chapel Hill, NC 27599, USA; 3 Department of Pharmacology, School of Medicine, College of Medicine, Taipei Medical University, Taipei 100301, Taiwan; fllin@tmu.edu.tw (F.-L.L.); geohsiao@tmu.edu.tw (G.H.); 4 Ph.D. Program in Drug Discovery and Development Industry, College of Pharmacy, Taipei Medical University, Taipei 100301, Taiwan; 5 Biomedical Commercialization Center, Taipei Medical University, Taipei 100301, Taiwan

**Keywords:** HDAC inhibitor, HSP90, blue light, neovascularization, pharmacophore, scaffold

## Abstract

Age-related macular degeneration (AMD) occurs due to an abnormality of retinal pigment epithelium (RPE) cells that leads to gradual degeneration of the macula. Currently, AMD drug pipelines are endowed with limited options, and anti-VEGF agents stand as the dominantly employed therapy. Despite the proven efficacy of such agents, the evidenced side effects associated with their use underscore the need to elucidate other mechanisms involved and identify additional molecular targets for the sake of therapy improvement. The previous literature provided us with a solid rationale to preliminarily explore the potential of selective HDAC6 and HSP90 inhibitors to treat wet AMD. Rather than furnishing single-target agents (either HDAC6 or HSP90 inhibitor), this study recruited scaffolds endowed with the ability to concomitantly modulate both targets (HDAC6 and HSP90) for exploration. This plan was anticipated to accomplish the important goal of extracting amplified benefits via dual inhibition (HDAC6/HSP90) in wet AMD. As a result, G570 (indoline-based hydroxamate), a dual selective HDAC6-HSP90 inhibitor exerting its effects at micromolar concentrations, was pinpointed in the present endeavor to attenuate blue light-induced cell migration and retinal neovascularization by inhibiting VEGF production. In addition to the identification of a potential chemical tool (G570), the outcome of this study validates the candidate HDAC6-HSP90 as a compelling target for the development of futuristic therapeutics for wet AMD.

## 1. Introduction

Age-related macular degeneration (AMD) occurs due to an abnormality of the retinal pigment epithelium (RPE) that leads to gradual degeneration of the macula [1,2,3]. Considered to be one of the leading causes of vision loss, AMD is broadly categorized into dry AMD and wet AMD (choroidal neovascularization-CNV). The former is characterized by the occurrence of drusen and geographic atrophy, while the latter is caused by the abnormal growth of blood vessels that leads to a consequent imbalance of the biochemical environment in the eye [4,5,6]. Previous studies have revealed that inflammation, oxidative stress, hypoxia, and angiogenesis are some of the critical processes involved in the progression of wet AMD [7,8]. In particular, vascular endothelial growth factor A (VEGF-A) is extensively implicated in CNV, and increased vascular permeability leads to the loss of vision. Recent explorations conducted in pursuit of elucidating the underlying mechanism involved in CNV further report that external stimuli that cause oxidative stress can increase VEGF production in RPE [9]. Recent imperative disclosures made in this context include the upregulation of VEGF expression by inflammatory factors in RPE cells, as well as hypoxia [10,11], along with the involvement of blue light-induced and laser-induced vascular regulatory factors in promoting CNV [12,13]. In summary, the neovascularization potential of VEGF plays a key role in the onset and progression of wet-form AMD. Accordingly, the currently available therapeutic options predominantly include the targeting of VEGF in the retina of patients.

In light of the aforementioned information, the efforts to date have been dominantly channelized toward the fabrication of anti-VEGF agents in pursuit of extracting conclusive therapeutic benefits in CNV. However, revelations regarding the therapeutic growth of anti-VEGF agents are slightly disappointing, as their advancement to clinical-stage evaluations has often been impaired by the evidenced ocular side effects [14,15]. These side effects might not seem to outweigh the efficacy of anti-VEGF drugs at this point; however, they do outweigh long-term patient management and public health. Therefore, the construction of mechanistically diverse classes of drugs endowed with efficacy for treating wet AMD without causing discomfort is urgently needed. On the other hand, developing an oral small molecule new drug will be much more welcome than an invasive intravitreal injection treatment. Studies ascertaining the involvement of epigenetic mechanisms in ocular development and disease coupled with investigations centered on exploring epigenetic therapies to treat ocular diseases have garnered significant attention from researchers in recent times [16,17]. Despite the availability of mechanistically diverse epigenetic therapies, the current trends in ocular drug discovery have been mostly inclined toward the exhaustive exploration of HDAC inhibitors [18,19,20]. Most likely, the flexible and modular nature of the HDAC inhibitory pharmacophore that allows a rapid buildup of libraries of chemical tools is the reason behind the biased attention of medicinal chemists working in tandem with ocular biologists toward this class of epigenetic therapies. In particular, selective inhibition of the HDAC6 isoform has been deduced to be a pragmatic approach in light of the demonstrated efficacy of tubastatin in protecting against oxidative stress in a photoreceptor cell line [21]. Moreover, the study also reported that tubastatin A restores visual function in a zebrafish model of inherited blindness. In another study, tubatatin A was found to be a photoreceptor neuroprotectant and demonstrated promise as a therapeutic option for retinal and macular degeneration [22]. These notions indicate that the rational design and evaluation of a selective HDAC 6 inhibitor is an important step forward in this direction. However, our attempts to leverage the learnings from the iterative medicinal chemistry program (previous) of our laboratory made us well acquainted with the fact that an amplified bioactivity profile of the HDAC6 inhibitory pharmacophore can be attained via logical tetheration with a fragment capable of modulating a biochemically related target. To accomplish this task, we started digging into the literature to identify another relevant protein/enzyme/target and arrived at some valuable revelations that support the candidate HSP90 chaperone protein as another potential target for the treatment of retinal degeneration. As such, HSP90 is widely present in all retinal layers, including RPE cells, and the expression of HSP90 is significantly enhanced during the progression of AMD [23,24,25]. Moreover, an HSP90 inhibitor (geldanamycin) inhibits hypoxia-induced VEGF expression in RPE cells and demonstrates the potential to exert antineovascularization effects [26]. Collectively, the aforementioned notions led us to assume that HDAC6-HSP90 constitutes a compelling target for wet AMD therapy.

**Figure 1:**
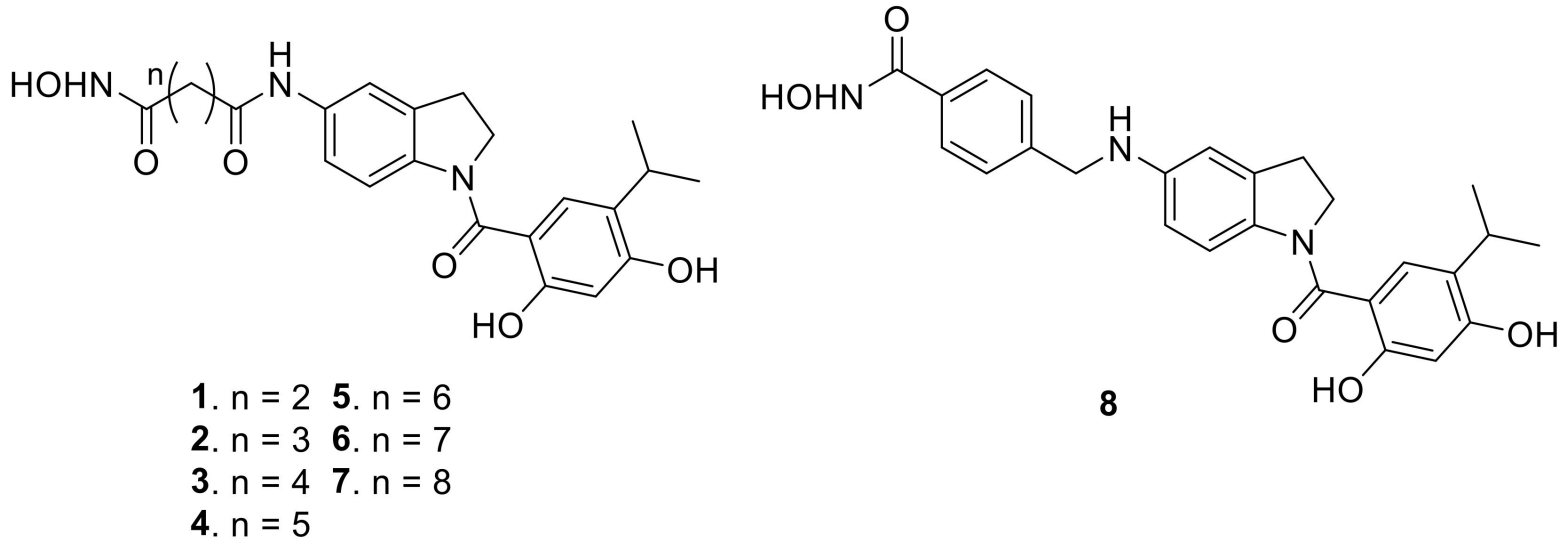
Structures of the designed compounds.

Intrigued, or rather stimulated, by the unjustified negligence of researchers in capitalizing on the benefits of dual HDAC6–HSP90 inhibition as a promising ocular therapeutic strategy, we believed that the task of developing therapeutics for wet AMD should involve the construction of chemical tools that can simultaneously inhibit HDAC enzymes and HSP90 chaperone proteins. Despite this strong hypothesis, designing, synthesizing, and developing a successful dual inhibitor is far from straightforward, and the translation from concept to practice, at times, can be very challenging. Fortunately, and much to our delight, the synthetic bank of our laboratory is presently loaded with several classes of hybrid scaffolds composed of HDAC inhibitory fragments (partially hydrogenated heteroaryl) [27,28] and HSP90 inhibitory pharmacophores (resorcinol fragments) [29,30,31] that were synthesized as a part of previous medicinal chemistry-based endeavors (anticancer drug discovery) (Figure 1) [32]. In the quest to expand the therapeutic profile of the aforementioned chemical architectures, we evaluated the activity profile of the series of indoline-based hydroxamates for the treatment of wet AMD in this study. As a result, a potent scaffold (G570) exerting inhibitory effects toward HDAC6 and HSP90 at nanomolar concentrations was identified as a promising compound that could attenuate blue light-induced migration in RPE cells and neovascularization in mice through decreased VEGF production.

## 2. Results

### 2.1. Synthesis of the Small Molecules

**Scheme 1:**
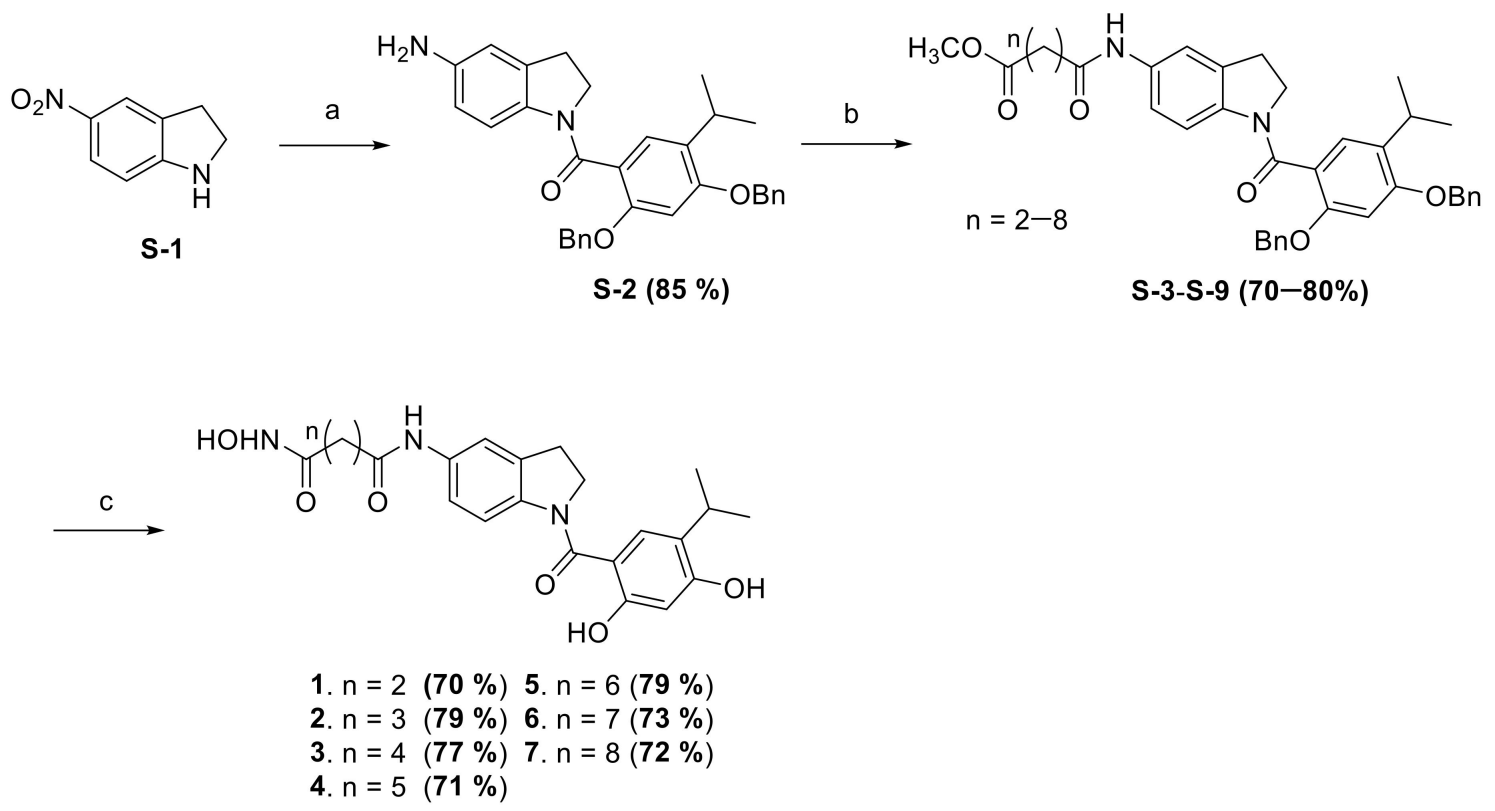
Reagents and conditions (**a**) (i) 2,4-bis(benzyloxy)-5-isopropylbenzoyl chloride, TEA, DCM, stirring, rt; (ii) Fe, NH_4_Cl, C_2_H_5_OH, reflux; (**b**) alkoxyalkanoic acid, EDC, HOBt, DIPEA, DMF, stirring, rt; (**c**) (i) LiOH, stirring, rt; (ii) NH_2_OBN, EDC, HOBt, DIPEA, DMF, stirring, rt; (iii) Pd/C, H_2_, stirring, rt.

**Scheme 2:**
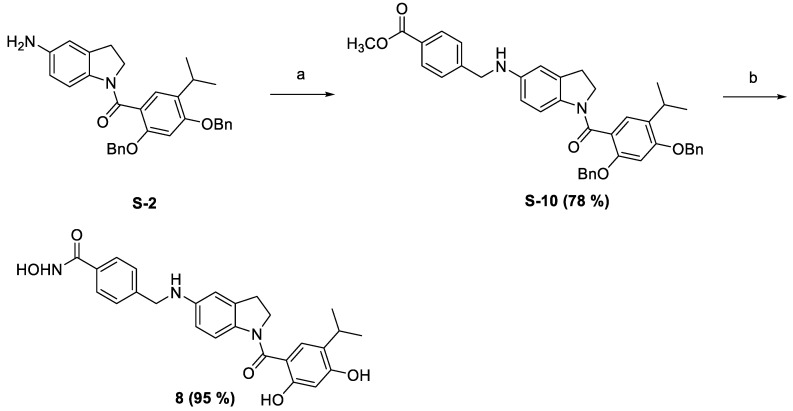
Reagents and conditions (**a**) methyl 4-formylbenzoate, NaCNBH_3_, methanol, stirring, rt; (**b**) (i) LiOH, stirring, rt; (ii) NH_2_OBn, EDC, HOBt, DIPEA, DMF, stirring, rt; (iii) BCl_3_, DCM, 0 °C.

The routes to the synthesis of the target compounds are illustrated in Scheme 1 and Scheme 2. The synthesis of the long-chain linker bearing hydroxamic acids commenced with the reaction of 5-nitroindoline and 2,4-bis(benzyloxy)-5-isopropylbenzoyl chloride to yield an intermediate that was subjected to nitro reduction using Fe/NH_4_Cl. The amine (**S-2**) underwent amidation with alkoxyalkanoic acids of varied length employing EDC/HOBt assisted methodology to generate intermediates **S-3** to **S-9**. Lithium hydroxide-mediated hydrolysis of the esters (**S-3**-**S-9**) yielded carboxylic acids, which were subsequently amidated with NH_2_OBn followed by debenzylation with Pd/C under a hydrogen atmosphere to furnish the target compounds (**1**–**7**). To further evaluate the impact of linker variation on the activity profile, construct 8 containing a benzyl linker between the indoline skeleton and hydroxamic acid was furnished as per the methodology depicted in Scheme 2. Scheme 2 initiated with reductive amination of methyl 4-formylbenzoate employing intermediate **S-2** to generate the adduct (**S-10**). Ester hydrolysis of **S-10** to generate carboxylic acid followed by EDC/HOBt-assisted amidation and BCl_3_-mediated debenzylation produced the target compound (**8**).

### 2.2. Inhibitory Activity of the Synthesized Small Molecules

The enzyme inhibitory potential of the synthesized compounds was evaluated against HeLa nuclear extract as the HDAC source and HSP90 (Table 1). Careful observation of Table 1 indicates that all the compounds were able to remarkably inhibit the HSP90 chaperone with IC_50_ values ranging from 49 to 85 nM; however, HeLa HDAC did not exhibit any sensitivity to compound **1** bearing a short linker (long-chain alkyl linker bearing compound, *n* = 2) and the benzyl linker containing compound (compound **8**). Overall, compound **4** and compound **5** were identified as the most potent inhibitors of HeLa HDAC as well as HSP90. The compounds were further evaluated for HDAC isoform inhibitory effects against HDAC1, 3, 6, and 8 (Table 2). Trichostatin A was employed as the standard. Subtle variations in the linker region (tethering indoline core with hydroxamic acid) had an impact on the HDAC isoform inhibitory activity. In general, all the compounds demonstrated a striking selective inhibitory profile toward the HDAC6 isoform, with compounds **2**, **4**, **5**, and **6** inhibiting the HDAC6 isoform at single-digit nanomolar concentrations. Among them, compound **5** (**570**), bearing the six methylene chain linker, was identified as the most potent inhibitor with an IC_50_ value of 1.15 nM and exhibited a selectivity ratio of **113** for HDAC1/HDAC6, **139** for HDAC3/HDAC6, and **246** for HDAC8/HDAC6. In view of the balanced HSP90 and HDAC6 inhibitory profile of compound **5** (G570), exhaustive exploration of G570 was planned, and the results are presented in the following sections.

### 2.3. Effect of G570 and Blue Light (BL) Exposure on the Viability of ARPE-19 Cells

**Figure 2:**
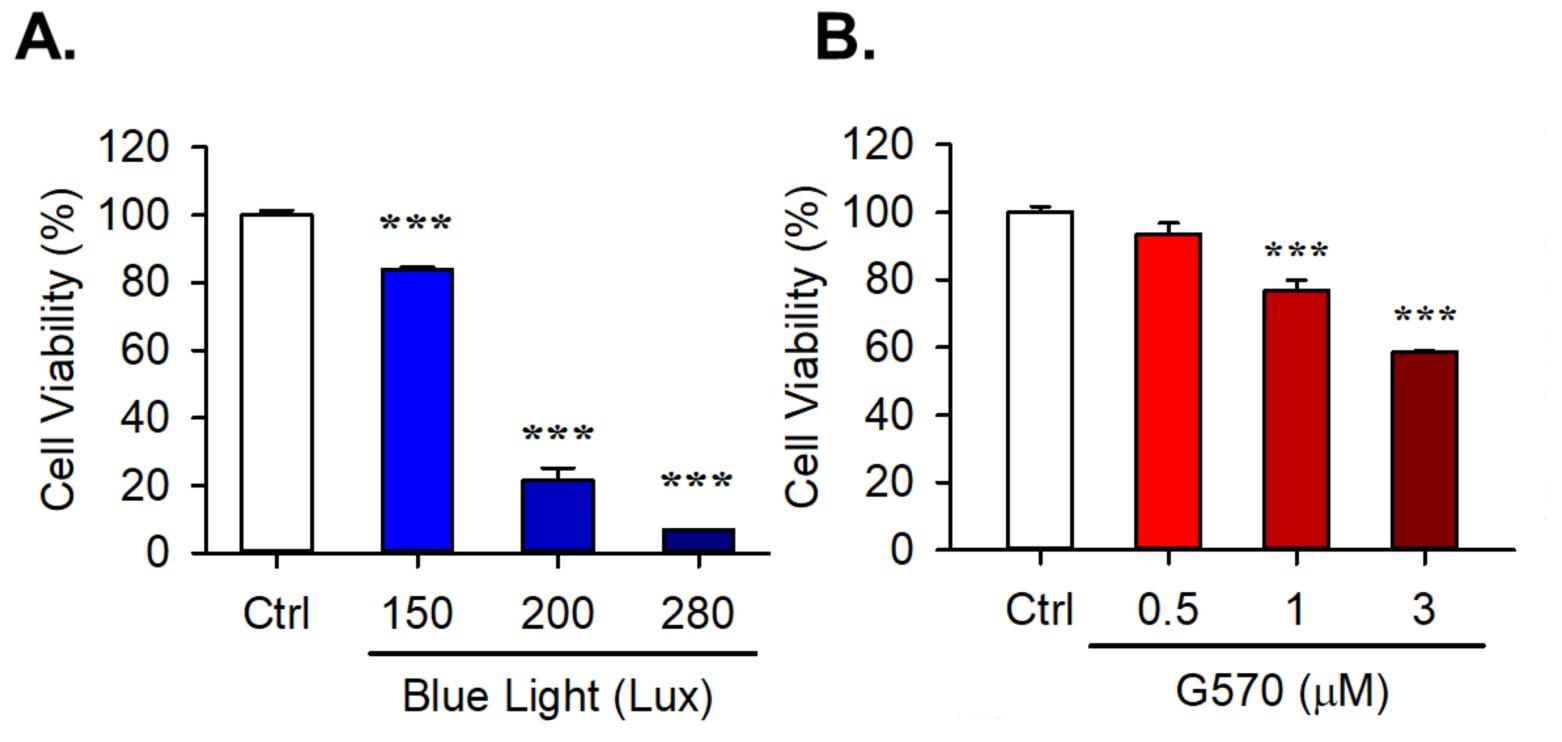
Cell viability of ARPE-19 cells. The cells were seeded in a 48-well plate. The next day, they were treated with varying intensities of BL (**A**) and concentrations of G570 (**B**). After 24 h, the MTT assay was performed to determine cell viability. Data are expressed as the mean ± S.D. and were obtained in triplicates. Significance was estimated compared to the control, where *** ≤ 0.001.

To obtain the optimal concentration of G570, the study drug and the optimal intensity of blue light were tested in ARPE-19 cells. We analyzed the effect of varying drug concentrations and BL intensities on cell viability. The results showed that 150, 200, and 280 lux BL decreased cell viability by 16.21 ± 0.76%, 78.46 ± 3.62%, and 93.14 ± 0.09%, respectively (Figure 2A). Upon treating cells with G570 at varying concentrations of 0.5, 1, and 3 µM for 24 h, the cell viability was found to be up to 93.38 ± 3.46%, 76.90 ± 3.03%, and 58.63 ± 0.56% (Figure 2B). However, the group treated with 0.5 µM maintained viability up to 93.38 ± 3.46% and showed the maximum level of activity in our preliminary analysis. Thus, in the following in vitro experiments, this concentration of G570 was used.

### 2.4. Effect of G570 on BL-Induced Migration in ARPE-19 Cells

**Figure 3:**
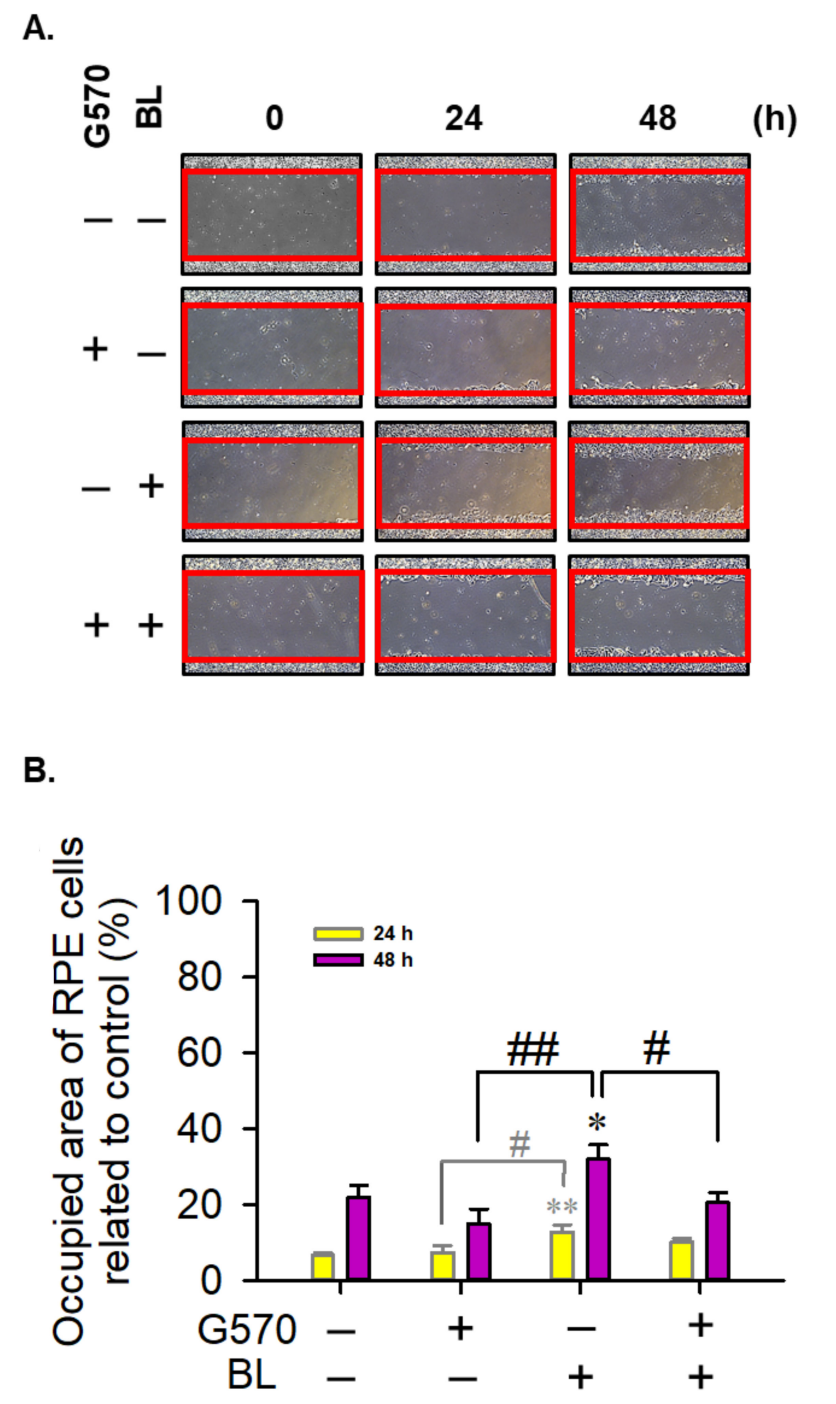
Cell migration assay in ARPE-19 cells. The cells were seeded in a 3.5 cm dish. Each group was exposed to 150 lux BL and treated with 0.5 μM G570. After 24 and 48 h, ARPE-19 cells were observed and recorded by an Olympus IX70 inverted tissue culture microscope and an SGHD-3.6C charge-coupled device (CCD) imaging system (SAGE Vision, New Taipei City, R.O. C), respectively. (**A**) The cell migration images of ARPE-19 cells. (**B**) The occupied area of ARPE-19 cells in different groups. Data are expressed as the mean ± S.D. and were obtained in triplicates. Significance was estimated compared to the control, where *p* * ≤ 0.05, *p* ** ≤ 0.01, *p* ^#^ ≤ 0.05, and *p* ^##^ ≤ 0.01.

From the literature reports, BL regulates cell function by inducing oxidative stress and inflammatory factors [33,34]. To investigate the effects of G570 on the migration of BL-irradiated ARPE-19 cells, we used a wound scratch model to evaluate the cell migration ability after different exposure durations of BL. The results showed that 150 lux BL at 24 h and 48 h increased cell migration, and G570 (0.5 µM) significantly inhibited this effect. (Figure 3A). The quantitative analysis is shown in Figure 3B. BL exposure for 24 h and 48 h significantly increased cell migration to 12.74 ± 1.90% and 31.97 ± 3.80%, respectively. When cotreated with G570 (0.5 µM), the migration was decreased to 10.05 ± 0.98% and 20.52 ± 2.60% (Figure 3B).

### 2.5. Effect of G570 on VEGF-189 and Its Receptor in BL-Exposed ARPE-19 Cells

**Figure 4:**
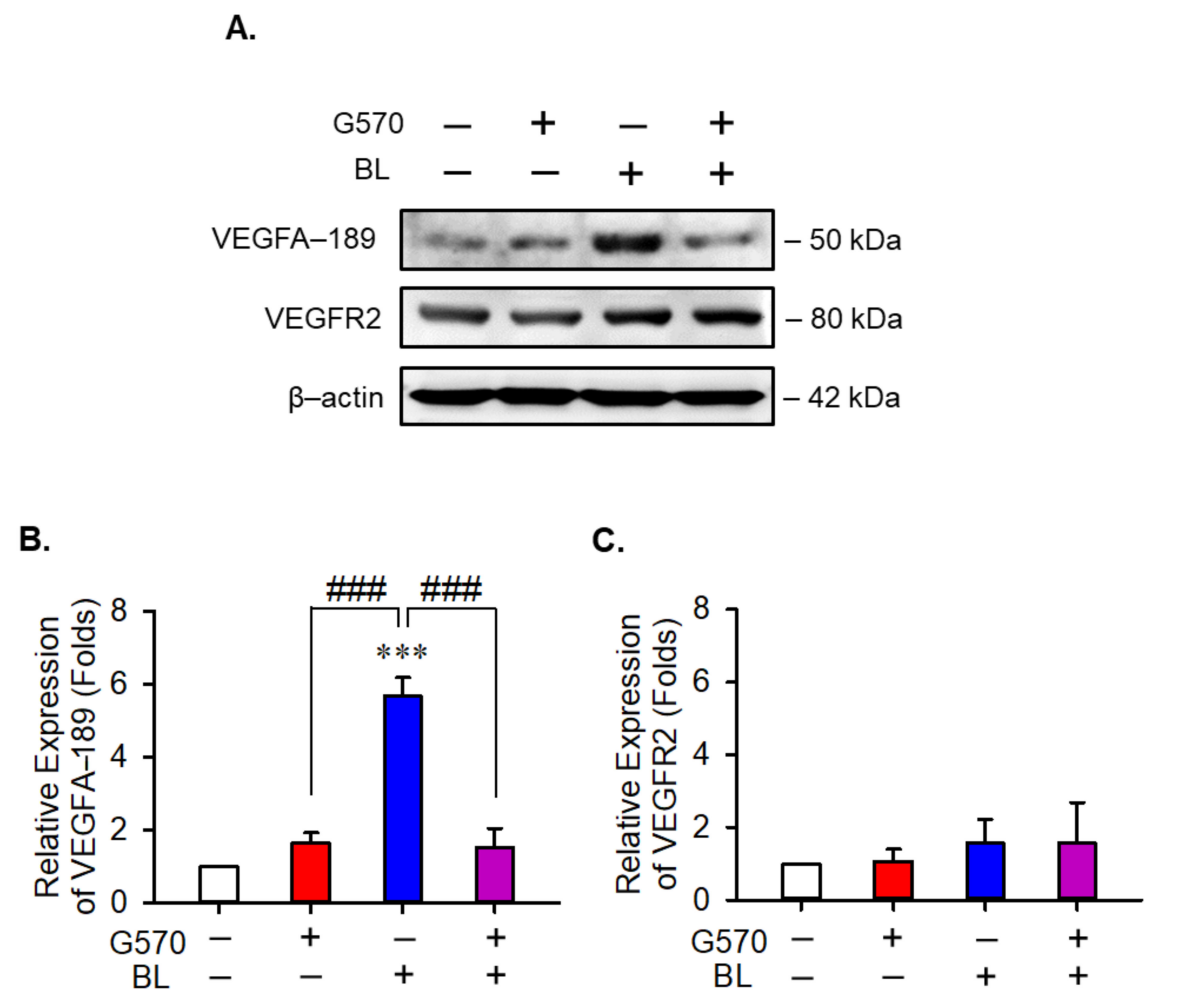
Expression of VEGFA-189 and VEGFR2 in BL-exposed ARPE-19 cells. The cells were seeded in a 6 cm dish. Each group was exposed to 150 lux BL and treated with 0.5 µM G570. After 24 h, the cells were collected, and total protein was isolated for the western blot assay. (**A**) The expression levels of VEGFA-189 and VEGFR2 in ARPE-19 cells. (**B**,**C**) represent the densitometry of (**A**). Data are expressed as the mean ± S.D. and were obtained in triplicates. Significance was estimated compared to the control, where *p* *** ≤ 0.001 and *p* ^###^ ≤ 0.001.

Studies have reported that VEGF is one of the most crucial proteins in wet-form AMD [35], therefore making it a key biomarker. To observe the effect of G570 on the expression levels of VEGFA-189 and VEGFR2, we performed Western blotting. The results showed that G570 significantly decreased the expression levels of VEGFA-189 by 4.15-fold in the presence of BL (Figure 4A,B). However, no significant change in the expression levels of VEGFR2 was observed in the presence of BL, G570, or both (Figure 4A,C). These results clearly indicate that even though G570 is not directly targeted to VEGF, it displays remarkable efficacy. Following this, we wanted to explore the VEGF-related pathway in detail.

### 2.6. Effect of G570 on the Cell Migration and Proliferation Pathway of Angiogenesis in BL-Exposed ARPE-19 Cells

**Figure 5:**
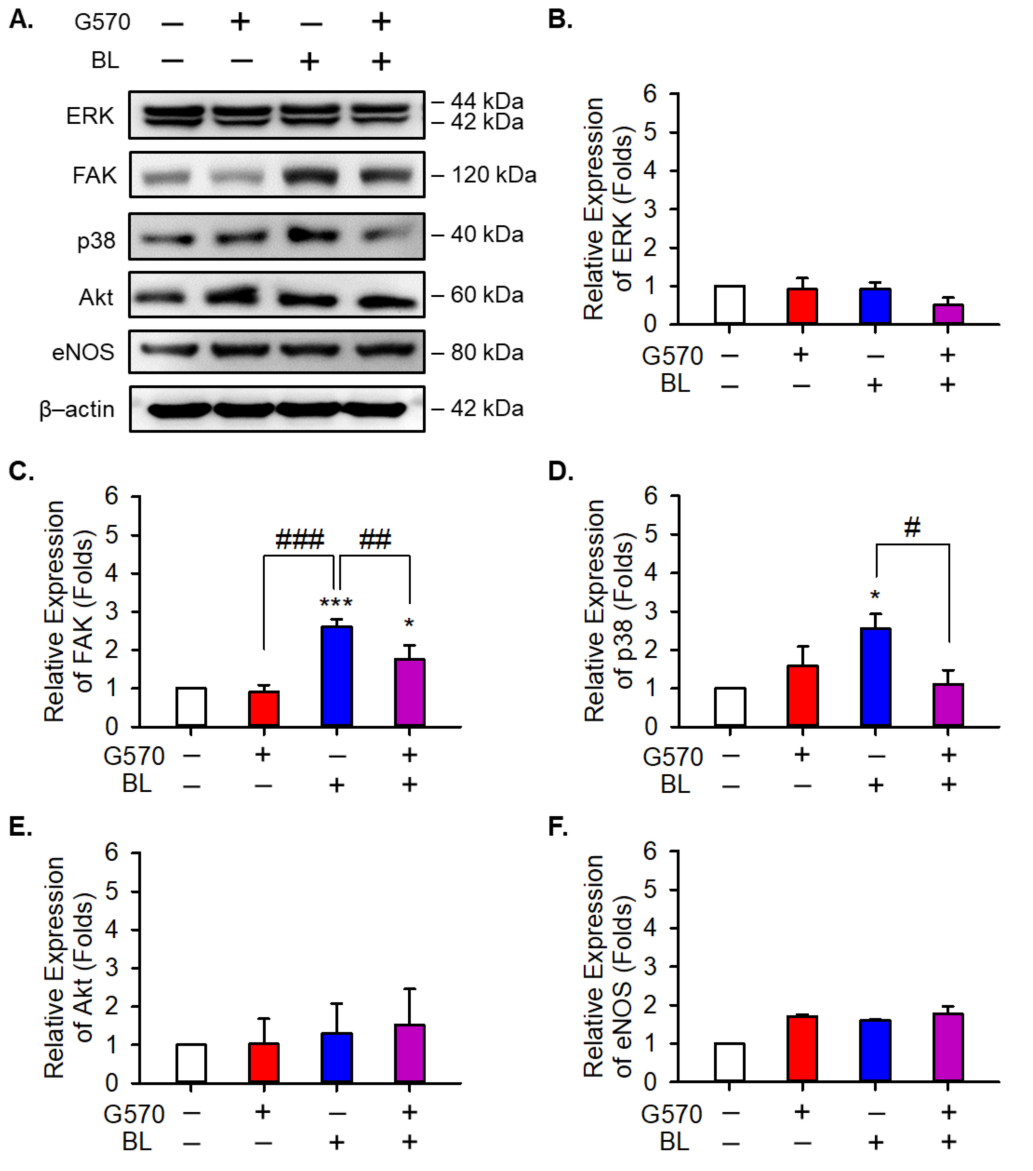
Expression of VEGFA-related proteins in BL-exposed ARPE-19 cells. The cells were seeded in a 6 cm dish. Each group was exposed to 150 lux BL and treated with 0.5 µM G570. After 24 h, the cells were collected, and total protein was isolated for the western blot assay. (**A**) Western blot of VEGFA-related proteins in ARPE-19 cells. Densitometric analysis of (**A**) was performed to obtain quantitative values for the expression levels of (**B**) ERK, (**C**) FAK, (**D**) p38, (**E**) Akt, and (**F**) eNOS in various treatment groups. Data are expressed as the mean ± S.D. and were obtained in triplicates. Significance was estimated compared to the control, where *p* * ≤ 0.05, *p* *** ≤ 0.001; *p* ^#^ ≤ 0.05; *p* ^##^ ≤ 0.01, and *p* ^###^ ≤ 0.001.

Angiogenesis takes place in wet-form AMD, and the onset of angiogenesis takes place through robust increases in the occurrence of various cellular processes, such as proliferation, migration, and permeability. Therefore, we wanted to see the effect of G570 on the proteins associated with these cellular processes. Our results showed that the expression levels of FAK, the protein associated with cell migration, increased significantly up to 2.61-fold due to BL exposure. When cells were treated with G570 in BL exposure, there was a significant decrease of 0.86-fold in the expression levels of FAK (Figure 5C). Analysis of the effect of G570 on p38, a protein also associated with cell migration, showed that G570 was able to attenuate the increase in the expression levels caused by BL by a significant 1.44-fold (Figure 5D). G570 had no considerable effect on ERK, eNOS, and Akt, proteins associated with cell proliferation and permeability (Figure 5B,E,F). Therefore, G570 interacts with cell migration and has a significant capability to intervene in angiogenesis through cell migration.

### 2.7. Effect of G570 on Laser-Induced Choroidal Neovascularization (CNV) in C57BL/6 Mice

**Figure 6:**
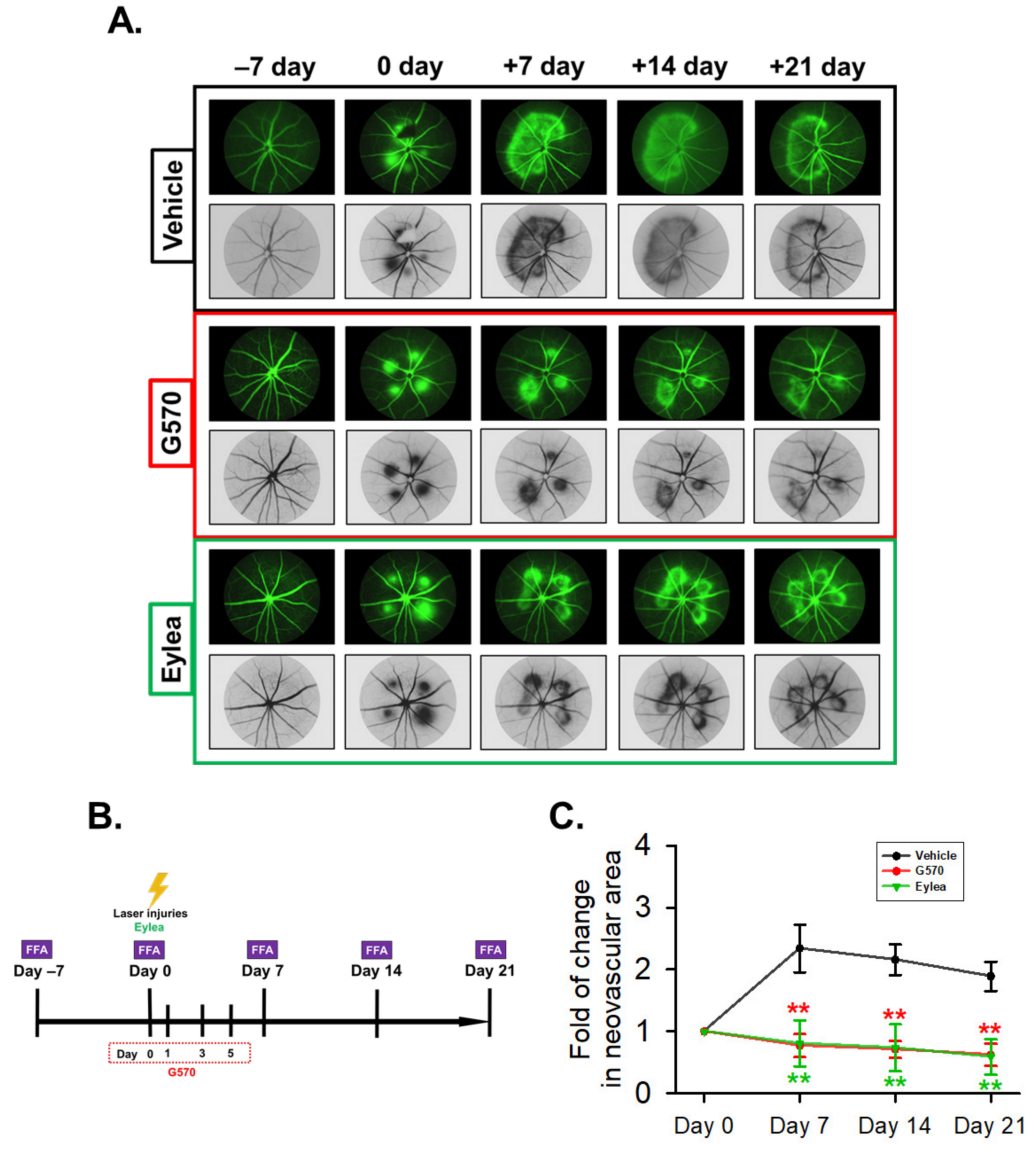
Effect of G570 in Laser-induced CNV model. A healthy profile of C57BL/6 mice was recorded by FFA 7 days before laser injury. Laser injury was executed on day 0. After laser injury, G570 was intraperitoneally administered (20 mg/kg per day) on days 0, 1, 3, and 5. The Eylea group, which served as the positive control, was treated by intravitreal injection (40 μg/1 μL per eye) on day 0. FFA was recorded at days −7, 0, 7, 14, and 21. (**A**) FFA figures of C57BL/6 mice. (**B**) Flow chart of laser-induced CNV experiment. (**C**) Fluorescein leakage fold of each group. The leakage area was evaluated. Data are expressed as the mean ± S.D. and were obtained in triplicates. Significance was estimated compared to the vehicle. *p* ** ≤ 0.01.

We wanted to validate our in vitro study by confirming the role of VEGF in neovascularization in animals. Therefore, we used a laser-induced CNV model to study the efficacy of G570 by analyzing fundus fluorescein angiography (FFA). Eylea was used as the positive control, and is an antiangiogenic therapy that tightly binds to all isoforms of VEGF-A and PlGF. Our data showed that Eylea significantly decreased the laser-induced leakage area of fluorescein starting from day 7 compared to the vehicle group. After treating the mice with G570 at days 0, 1, 3, and 5, a significant decrease in the area of neovascularization, as well as the leakage of fluorescein, was observed at day 7 (Figure 6A,B). When comparing the images, it was clearly visible that treatment with G570 ameliorated the neovascularization caused by the laser. Similarly, the comparison between the vehicle group and G570 group on day 21 also showed a significant decrease in both the neovascularization area as well as the leakage.

## 3. Discussion

Since there are limited therapeutic options for the treatment of CNV, the drug pipeline for the treatment of this ocular disease must be loaded with mechanistically diverse agents. Aligned with this objective, the present study addresses the exploration of logically constructed indoline-based scaffolds to treat wet AMD. The structural template of the target compounds comprises core structural units reported to inhibit the HDAC6 isoform and HSP90 chaperone. Among the hydroxamate series, one of the adducts (G570) demonstrated significant efficacy in attenuating blue light induced migration in RPE cells and laser-induced neovascularization in mice through decreased VEGF production. Notably, G570 is a potent inhibitor of the HDAC6 isoform and HSP90, as evident from the results of the enzymatic assays. These notions support the hypothesis of this research work and present dual selective HDAC6/HSP90 inhibition as a potential approach to extract therapeutic benefits in wet AMD.

VEGF plays a vital role in vascularization and angiogenesis, and is also a key inducer of vascular permeability. In humans, it has four major isoforms: VEGFA-121, -165, -189, and -206. However, only VEGFA-165 and VEGFA-189 induce neovascularization, and VEGFA-189 is expressed in highly branched neovasculature [36]. Our results showed a significant increase in the expression of VEGFA-189 due to blue light exposure, which can correlate with the induction of highly branched neovasculature. However, G570 was able to alleviate this increase significantly, as shown in Figure 6A. As mentioned earlier, there are several drugs on the market that directly target VEGF—such as pegaptanib sodium, bevacizumab, ranibizumab, and the most recently developed agent, aflibercept (Eylea). Pegaptanib sodium specifically targets VEGFA-165. However, bevacizumab and ranibizumab interact with all VEGFA isoforms. Eylea inhibits all VEGFA isoforms as well as placental growth factors (PlGF). However, they present associated side effects, such as macular degeneration, retinal hemorrhage, conjunctival hemorrhage, intraocular inflammation, and endophthalmitis. There is also a decrease in the efficacy of these drugs after long-term treatment. In addition, these anti-VEGF agents do not have enough efficacy in 50% of patients due to unknown reasons.

FAK is also a key marker in this study, and it has been reported that FAK deficiency affects cell survival, migration, and proliferation. Several angiogenic growth factors—such as insulin-like growth factor-I, angiopoietin-1, essential fibroblast growth factor, VEGF, and TNF-related activation-related cytokines—are significantly coupled with the activation of FAK [37]. Our results showed a similar trend: G570 significantly decreased the expression levels of VEGF-189 and FAK induced by blue light exposure (Figure 4 and Figure 5). A previous study reported that FAK activity controls the transcription of VEGFR2 [38]. However, our results did not show any significant correlation between the upregulation of FAK and VEGFR2. Additionally, the expression levels of VEGFR2 were not responsive to G570 treatment. The difference in these findings can be explained by the use of the different cell models or the inducer.

In ocular therapeutics, macromolecules with a radius of more than 10 Å are excluded by epithelial permeability, limiting intracorneal drug delivery [39]. Therefore, anti-VEGF drugs that are mainly designed to bind with VEGF, which are macromolecules, are delivered by invasive methods such as intravitreal injections [40]. This points to the dire need for noninvasive methods of delivery, and for that, developing small molecules with high efficacy is required. These molecules will overcome shortcomings such as bioavailability and targeting. G570 is an HDAC/HSP90 inhibitor and presents anti-VEGF activity, which, if studied and explored extensively, can open various avenues of drug development against wet-form AMD and can also show significant activity without the associated complications. G570 also possesses an edge over current drugs due to its size, and is a small molecule; therefore, it can penetrate the cornea. In summary, these results provide sufficient evidence that G570 has therapeutic potential against wet-form AMD.

## 4. Materials and Methods

### 4.1. Synthesis of the Compounds 

Nuclear magnetic resonance (NMR) spectra were obtained with Bruker DRX-500 spectrometer (operating at 300 MHz), and chemical shifts are cited in parts per million (ppm, d) downfield from TMS as an internal standard. High-resolution mass spectra (HRMS) were measured with a JEOL (JMS-700) electron impact (EI) mass spectrometer. The purity of the final compounds was determined using an Agilent 1100 series HPLC system using a C-18 column (Agilent ZORBAX Eclipse XDB-C18 5 μm, 4.6 × 150 mm). All compounds were found to have a purity of ≥95%. Flash column chromatography was done using silica gel (Merck Kieselgel 60, No. 9385, 230e400 mesh ASTM). All reactions were carried out under an atmosphere of dry N_2_ (Supplementary materials).

#### 4.1.1. Synthesis of (5-Aminoindolin-1-yl)(2,4-bis(benzyloxy)-5-isopropylphenyl)methanone (**S-2**)

2,4-Bis(benzyloxy)-5-isopropylbenzoic acid (2 g, 5.31 mmol) was dissolved in CH_2_Cl_2_ (10 mL), and oxalyl chloride (2 mL) was added. The reaction mixture was stirred for 3 h at room temperature, and then the solvent was evaporated under reduced pressure to give a solid residue. This residue was dissolved in CH_2_Cl_2_ (10 mL), and 5-nitroindoline (0.872 g, 5.31 mmol) was added, followed by Et_3_N (1.86 mL, 13.2 mmol). The reaction mixture was stirred for 12 h and then quenched with H_2_O. CH_2_Cl_2_ (50 mL × 3) was used for the extraction. The combined organic layer was dried over anhydrous MgSO_4_ and concentrated under reduced pressure. The residue was purified by silica gel chromatography. To the solution of purified compound (1 g, 1.91 mmol) in EtOH (45 mL) and H_2_O (5 mL), iron powder (0.534 g, 9.57 mmol), and NH_4_Cl (0.204 g, 3.82 mmol) were added. The reaction mixture was refluxed for 5 h, and the progress of the reaction was monitored on TLC. On completion of the reaction, the reaction was filtered over celite. The filtrate was concentrated, and H_2_O was added to it. Extraction was done with EtOAc (50 mL × 3). The combined organic layer was dried over anhydrous MgSO_4_ and concentrated under reduced pressure. The resulting residue was purified by silica gel chromatography (n-hexane:EtOAc = 3:2) to give compound **S-2** in 85% yield; ^1^HNMR (300 MHz, CD_3_OD): 7.27–7.69 (m, 13H), 6.79 (d, *J* = 7.2 Hz, 1H), 6.61 (s, 1H), 5.16 (s, 4H), 4.01 (t, *J* = 7.2 Hz, 2H), 3.11 (m, 1H), 3.01 (t, *J* = 8.1 Hz, 2H), 1.12 (d, *J* = 6.9 Hz, 6H).

#### 4.1.2. Synthesis of Methyl 4-((1-(2,4-Bis(benzyloxy)-5-isopropylbenzoyl)indolin-5-yl)amino)-4-oxobutanoate (**S-3**)

A mixture of **S-2** (0.3 g, 0.609 mmol), EDC.HCl (0.232 g, 1.21 mmol), HOBt (0.123 g, 737 mg, 0.914 mmol), 4-methoxy-4-oxobutanoic acid (0.96 g, 0.727 mmol) and DIPEA (0.265 mL, 1.52 mmol) in DMF (5 mL) was stirred at rt for 5 h. After being stirred for a further 5 h, the reaction mixture was quenched with H_2_O and extracted with EtOAc (50 mL × 3). The combined organic layer was dried over anhydrous MgSO_4_, concentrated under reduced pressure and purified by silica gel chromatography (hexane:EtOAc = 4:1) to give **S-3** in 80% yield; ^1^HNMR (300 MHz, CDCl_3_): 7.24–7.69 (m, 12H), 6.97–7.07 (m, 2H), 6.55 (s, 1H), 5.06 (s, 4H), 4.01 (t, *J* = 7.2 Hz, 2H), 3.69 (s, 3H), 3.11 (m, 1H), 3.01 (t, *J* = 8.1 Hz, 2H), 2.34 (bs, 4H), 1.14 (d, *J* = 6.9 Hz, 6H).

#### 4.1.3. Synthesis of Methyl 5-((1-(2,4-Bis(benzyloxy)-5-isopropylbenzoyl)indolin-5-yl)amino)-5-oxopentanoate (**S-4**)

The title compound (**S-4**) was synthesized in 72% yield using (5-aminoindolin-1-yl)(2,4-bis(benzyloxy)-5-isopropylphenyl)methanone and 5-methoxy-5-oxopentanoic acid in a manner similar to that described for the synthesis of compound **S-3**; ^1^HNMR (300 MHz, CDCl_3_): 7.21–7.62 (m, 12H), 6.92–7.01 (m, 2H), 6.52 (s, 1H), 5.09 (s, 4H), 3.99 (t, *J* = 7.2 Hz, 2H), 3.71 (s, 3H), 3.13 (m, 1H), 3.0 (t, *J* = 8.1 Hz, 2H), 2.24–2.31 (m, 4H), 1.52 (m, 2H), 1.12 (d, *J* = 6.9 Hz, 6H).

#### 4.1.4. Synthesis of Methyl 6-((1-(2,4-Bis(benzyloxy)-5-isopropylbenzoyl)indolin-5-yl)amino)-6-oxohexanoate (**S-5**)

The title compound (**S-5**) was synthesized in 78% yield using (5-aminoindolin-1-yl)(2,4-bis(benzyloxy)-5-isopropylphenyl)methanone and 6-methoxy-6-oxohexanoic acid in a manner similar to that described for the synthesis of compound **S-3**; ^1^HNMR (300 MHz, CDCl_3_): 7.24–7.59 (m, 12H), 6.92–6.99 (m, 2H), 6.54 (s, 1H), 5.07 (s, 4H), 3.97 (t, *J* = 7.2 Hz, 2H), 3.65 (s, 3H), 3.00-3.11 (m, 3H), 2.29 (m, 4H), 1.52–1.54 (m, 4H), 1.14 (d, *J* = 6.9 Hz, 6H).

#### 4.1.5. Synthesis of Methyl 7-((1-(2,4-Bis(benzyloxy)-5-isopropylbenzoyl)indolin-5-yl)amino)-7-oxohexanoate (**S-6**)

The title compound (**S-6**) was synthesized in 74% yield using (5-aminoindolin-1-yl)(2,4-bis(benzyloxy)-5-isopropylphenyl)methanone and 7-methoxy-7-oxoheptanoic acid in a manner similar to that described for the synthesis of compound **S-3**; ^1^HNMR (300 MHz, CDCl_3_): 7.23–7.54 (m, 12H), 6.92–7.02 (m, 2H), 6.59 (s, 1H), 5.11 (s, 4H), 3.98 (t, *J* = 7.2 Hz, 2H), 3.69 (s, 3H), 3.02–3.13 (m, 3H), 2.31 (t, *J* = 7.2 Hz, 2H), 2.22 (t, *J* = 6.3 Hz, 2H), 1.62–1.71 (m, 4 H), 1.41 (m, 2H), 1.21 (d, *J* = 6.9 Hz, 6H).

#### 4.1.6. Synthesis of Methyl 8-((1-(2,4-Bis(benzyloxy)-5-isopropylbenzoyl)indolin-5-yl)amino)-8-oxohexanoate (**S-7**)

The title compound (**S-7**) was synthesized in 72% yield using (5-aminoindolin-1-yl)(2,4-bis(benzyloxy)-5-isopropylphenyl)methanone and 8-methoxy-8-oxooctanoic acid in a manner similar to that described for the synthesis of compound **S-3;** ^1^HNMR (300 MHz, CDCl_3_): 7.21–7.59 (m, 12H), 6.97 (m, 2H), 6.51 (s, 1H), 5.08 (s, 4H), 3.97 (t, *J* = 7.2 Hz, 2H), 3.63 (s, 3H), 3.04–3.19 (m, 3H), 2.32 (t, *J* = 7.2 Hz, 2H), 2.16 (t, *J* = 6.3 Hz, 2H), 1.68 (m, 4 H), 1.41–1.48 (m, 4H), 1.15 (d, *J* = 6.9 Hz, 6H).

#### 4.1.7. Synthesis of Methyl 9-((1-(2,4-Bis(benzyloxy)-5-isopropylbenzoyl)indolin-5-yl)amino)-9-oxohexanoate (**S-8**)

The title compound (**S-8**) was synthesized in 76% yield using (5-aminoindolin-1-yl)(2,4-bis(benzyloxy)-5-isopropylphenyl)methanone and 9-methoxy-9-oxononanoic acid in a manner similar to that described for the synthesis of compound **S-3**; ^1^HNMR (300 MHz, CDCl_3_): 7.31–7.55 (m, 12H), 6.97 (m, 2H), 6.61 (s, 1H), 5.05 (s, 4H), 3.99 (t, *J* = 7.2 Hz, 2H), 3.62 (s, 3H), 3.05–3.14 (m, 3H), 2.38 (t, *J* = 7.2 Hz, 2H), 2.27 (t, *J* = 6.3 Hz, 2H), 1.62–1.71 (m, 4 H), 1.42–1.49 (m, 6H), 1.21 (d, *J* = 6.9 Hz, 6H).

#### 4.1.8. Synthesis of Methyl 10-((1-(2,4-Bis(benzyloxy)-5-isopropylbenzoyl)indolin-5-yl)amino)-10-oxohexanoate (**S-9**)

The title compound (**S-9**) was synthesized in 78% yield using (5-aminoindolin-1-yl)(2,4-bis(benzyloxy)-5-isopropylphenyl)methanone and 10-methoxy-10-oxodecanoic acid in a manner similar to that described for the synthesis of compound **S-3**; ^1^HNMR (300 MHz, CDCl_3_): 7.27–7.59 (m, 12H), 6.98–7.06 (m, 2H), 6.65 (s, 1H), 5.04 (s, 4H), 3.96 (t, *J* = 7.2 Hz, 2H), 3.65 (s, 3H), 3.08–3.17 (m, 3H), 2.28 (t, *J* = 7.2 Hz, 2H), 2.12 (t, *J* = 6.3 Hz, 2H), 1.52 –1.54 (m, 4 H), 1.41–1.45 (m, 8H), 1.19 (d, *J* = 6.9 Hz, 6H).

#### 4.1.9. Synthesis of N1-(1-(2,4-dihydroxy-5-isopropylbenzoyl)indolin-5-yl)-N4-hydroxysuccinamide (**1**)

A mixture of **S-3** (0.3 g, 0.49 mmol), 1M LiOH aq. (3 mL), and dioxane (5 mL) was stirred at rt for 2 h. The reaction was concentrated under reduced pressure and H_2_O was added. The mixture was acidified with 3N HCl and extracted with EtOAc (50 mL × 3). The combined organic layer was dried over anhydrous MgSO_4_ and concentrated under reduced pressure to yield the acid in 96% yield. The acid was subjected to EDC/HOBt amidation with NH_2_OBn.HCl (1.1 eq) in a manner similar to that described for the synthesis of compound **S-3**. To the solution of amide (0.2 g, 0.286 mmol) in MeOH (10 mL), a catalytic amount of 10% palladium on carbon was added, and the reaction mixture was stirred for 2 h under hydrogen. The reaction mixture was filtered over celite, and the filtrate was dried in a vacuum and purified by silica gel chromatography (EtOAc) to give **1** in 70% yield; mp: 206–207 °C. ^1^HNMR (300 MHz, DMSO-d_6_): 10.45 (1H), 9.91 (1H), 9.75 (1H), 9.63 (1H), 8.74 (1H), 7.57 (bs, 2H), 7.28 (d, *J* = 7.5 Hz, 1H), 6.99 (s, 1H), 6.42 (s, 1H), 3.97 (t, *J* = 8.1 Hz, 2H), 3.02–3.13 (m, 3H), 2.52 (bs, 2H), 2.30 (t, *J* = 6.3 Hz, 2H), 1.14 (d, *J* = 6.9 Hz, 6H); ^13^CNMR (75 MHz, DMSO-d_6_): 167.65, 166.23, 165.12, 154.52, 150.87, 136.04, 132.88, 130.81, 123.38, 115.12, 113.73, 112.98, 100.27, 46.66, 29.28, 25.33, 23.67, 20.47. HRMS (ESI) for C_22_H_24_N_3_O_6_ [M-H]^−^: calcd, 426.1665; found, 426.1663. 

#### 4.1.10. Synthesis of N1-(1-(2,4-dihydroxy-5-isopropylbenzoyl)indolin-5-yl)-N5-hydroxyglutaramide (**2**)

The title compound (**2**) was obtained in 79% yield in a manner similar to that described for the synthesis of compound **1**. mp: 200–201 °C. ^1^HNMR (300 MHz, DMSO-d_6_): 10.40 (s, 1H), 9.86 (s, 1H), 9.75 (s, 1H), 9.63 (s, 1H), 7.59 (bs, 2H), 7.27 (d, *J* = 7.8 Hz, 1H), 6.98 (s, 1H), 6.43 (s, 1H), 3.97 (t, *J* = 8.4 Hz, 2H), 3.02–3.13 (m, 3H), 2.30 (t, *J* = 7.5 Hz, 2H), 2.03 (t, *J* = 7.2 Hz, 2H), 1.81 (q, *J* = 7.2 Hz, 2H), 1.13 (d, *J* = 6.9 Hz, 6H); ^13^CNMR (75 MHz, DMSO-d_6_): 168.45, 166.56, 165.19, 155.59, 151.54, 134.01, 131.93, 132.76, 121.46, 114.21, 113.79, 111.76, 100.78, 45.62, 28.87, 24.46, 22.65, 22.57, 20.79. HRMS (ESI) for C_23_H_27_N_3_O_6_ (M + H^+^): calcd, 442.1978; found, 442.1982.

#### 4.1.11. Synthesis of N1-(1-(2,4-dihydroxy-5-isopropylbenzoyl)indolin-5-yl)-N6-hydroxyadipamide (**3**)

The title compound (**3**) was obtained in 77% yield in a manner similar to that described for the synthesis of compound **1.** mp: 190–191 °C. ^1^HNMR (300 MHz, DMSO-d_6_): 10.37 (s, 1H), 9.82 (s, 1H), 9.74 (bs, 2H), 8.69 (s, 1H), 7.59 (bs, 2H), 7.26 (d, *J* = 7.8 Hz, 1H), 6.99 (s, 1H), 6.41 (s, 1H), 3.97 (t, *J* = 8.1 Hz, 2H), 3.02–3.13 (m, 3H), 2.29 (bs, 2H), 1.97 (t, *J* = 6.3 Hz, 2H), 1.54–1.56 (m, 4 H), 1.13 (d, *J* = 6.9 Hz, 6H); ^13^CNMR (75 MHz, DMSO-d_6_): 168.59, 166.89, 165.12, 154.55, 150.89, 136.07, 132.86, 130.82, 123.39, 115.29, 113.91, 112.93, 100.28, 46.43, 30.00, 29.91, 23.69, 22.71, 22.63, 20.45. HRMS (ESI) for C_24_H_29_N_3_O_6_ [M-H]^−^: calcd, 454.1978; found, 454.1972.

#### 4.1.12. Synthesis of N1-(1-(2,4-dihydroxy-5-isopropylbenzoyl)indolin-5-yl)-N7-hydroxyheptanediamide (**4**)

The title compound (**4**) was obtained in 71% yield in a manner similar to that described for the synthesis of compound **1**. mp: 165–166 °C. ^1^HNMR (300 MHz, CD_3_OD): 7.57 (bs, 2H), 7.22 (d, *J* = 8.4 Hz, 1H), 7.14 (s, 1H), 6.40 (s, 1H), 4.13 (t, *J* = 8.1 Hz, 2H), 3.33 (m, 1H), 3.21 (t, *J* = 7.8 Hz, 2H), 2.38 (t, *J* = 7.2 Hz, 2H), 2.12 (t, *J* = 6.3 Hz, 2H), 1.68–1.72 (m, 4 H), 1.43 (bs, 2H), 1.20 (d, *J* = 6.9 Hz, 6H); ^13^CNMR (75 MHz, DMSO-d_6_): 169.54, 166.87, 166.27, 153.53, 151.43, 136.97, 131.32, 130.29, 122.43, 114.22, 113.43, 111.56, 100.45, 44.41, 30.11, 29.66, 23.22, 22.43, 22.31, 21.32, 20.45. HRMS (ESI) for C_25_H_31_N_3_O_6_ [M-H]^−^: calcd, 468.2135; found, 468.2135.

#### 4.1.13. Synthesis of N1-(1-(2,4-dihydroxy-5-isopropylbenzoyl)indolin-5-yl)-N_8_-hydroxyoctanediamide (**5**)

The title compound (**5**) was obtained in 79% yield in a manner similar to that described for the synthesis of compound **1.** mp: 125–126 °C. ^1^HNMR (300 MHz, DMSO-d_6_): 10.33 (s, 1H), 9.79 (s, 1H), 9.73 (s, 1H), 9.61 (s, 1H), 8.66 (d, *J* = 1.5 Hz, 1H),7.59 (bs, 2H), 7.25 (d, *J* = 7.5 Hz, 1H), 6.99 (s, 1H), 6.41 (s, 1H), 3.97 (t, *J* = 8.4 Hz, 2H), 3.02–3.08 (m, 3H), 2.28 (t, *J* = 7.2 Hz, 2H), 1.95 (t, *J* = 7.2 Hz, 2H), 1.48–1.60 (m, 4 H), 1.29 (bs, 4H), 1.13 (d, *J* = 7.2 Hz, 6H); ^13^CNMR (75 MHz, DMSO-d_6_): 168.19, 165.29, 164.32, 155.21, 151.34, 137.43, 131.99, 131.45, 130.54, 115.29, 113.48, 112.21, 99.17, 45.54, 34.65, 31.43, 25.31, 26.32, 23.48, 21.43. HRMS (ESI) for C_26_H_33_N_3_O_6_ [M-H]^−^: calcd, 482.2291; found, 482.2292.

#### 4.1.14. Synthesis of N1-(1-(2,4-dihydroxy-5-isopropylbenzoyl)indolin-5-yl)-N_9_-hydroxynonanediamide (**6**)

The title compound (**6**) was obtained in 73% yield in a manner similar to that described for the synthesis of compound **6**. mp: 110–111 °C. ^1^HNMR (300 MHz, DMSO-d_6_): 10.33 (s, 1H), 9.79 (s, 1H), 9.74 (s, 1H), 9.61 (s, 1H), 8.66 (bs, 1H), 7.59 (bs, 2H), 7.26 (d, *J* = 7.5 Hz, 1H), 6.99 (d, *J* = 2.7 Hz, 1H), 6.41 (d, *J* = 3.0 Hz, 1H), 3.97 (t, J = 8.4 Hz, 2H), 3.02–3.13 (m, 3H), 2.28 (t, J = 5.7 Hz, 2H), 1.95 (t, *J* = 6.9 Hz, 2H), 1.49–1.58 (m, 4 H), 1.29 (bs, 6H), 1.14 (d, *J* = 6.6 Hz, 6H); ^13^CNMR (75 MHz, DMSO-d_6_): 168.59, 166.89, 165.12, 154.54, 150.89, 136.04, 132.91, 130.79, 123.38, 115.25, 113.88, 112.96, 100.27, 46.69, 34.18, 30.08, 26.41, 26.29, 25.36, 23.68, 20.47. HRMS (ESI) for C_27_H_35_N_3_O_6_ [M-H]^−^: calcd, 496.2448; found, 496.2448.

#### 4.1.15. Synthesis of N1-(1-(2,4-dihydroxy-5-isopropylbenzoyl)indolin-5-yl)-N_10_-hydroxydecanediamide (**7**)

The title compound (**7**) was obtained in 72% yield in a manner similar to that described for the synthesis of compound **1**. mp: 100–101 °C. ^1^HNMR (300 MHz, DMSO-d_6_): 10.34 (s, 1H), 9.63–9.80 (m, 3H), 8.67 (bs, 1H), 7.59 (bs, 2H), 7.26 (d, *J* = 7.8 Hz, 1H), 6.99 (d, J = 2.4 Hz, 1H), 6.42 s,1H), 3.97 (t, *J* = 7.8 Hz, 2H), 3.02–3.13 (m, 3H), 2.28 (t, *J* = 6.9 Hz, 2H), 1.95 (t, *J* = 6.6 Hz, 2H), 1.49–1.58 (m, 4 H), 1.29 (bs, 8H), 1.14 (d, *J* = 6.6 Hz, 6H); ^13^CNMR (75 MHz, DMSO-d_6_): 169.84, 168.73, 166.97, 154.52, 150.89, 136.04, 132.92, 130.79, 123.38, 115.24, 113.87, 112.98, 100.26, 46.67, 34.19, 30.08, 26.55, 26.48, 23.67, 23.00, 22.92, 20.47, 18.87. HRMS (ESI) for C_28_H_37_N_3_O_6_ [M-H]^−^: calcd, 510.2604; 510.2605.

#### 4.1.16. Synthesis of Methyl 4-(((1-(2,4-Bis(benzyloxy)-5-isopropylbenzoyl)indolin-5-yAmino)methyl)benzoate (**S-10**)

To a stirred solution of (**S-2**) (0.500 g, 1.01 mmol) and methyl 4-formylbenzoate (0.166 g, 1.01 mmol) in EtOH (10 mL), a few drops of glacial AcOH were added. Sodium cyano-borohydride (0.095 g, 1.52 mmol) was added and the reaction mixture was stirred at room temperature overnight. The reaction was quenched with H_2_O and extracted with EtOAc. The combined organic layer was dried over anhydrous MgSO_4_, concentrated under reduced pressure. The residue was purified by silica gel chromatography (n-hexane:EtOAc = 3:2) to give compound **S-10** (yield 78%). ^1^HNMR (300 MHz, CDCl_3_): 7.59 (d, *J* = 7.8 Hz, 2H), 7.27–7.55 (m, 13H), 7.19 (s, 1H), 6.65 (s, 1H), 6.48 (d, *J* = 7.8 Hz, 1 H), 6.43 (s, 1H), 5.01 (s, 4H), 4.71 (s, 2H), 4.13 (t, *J* = 8.1 Hz, 2H), 3.81 (s, 3H), 3.08 (m, 1H), 3.02 (t, *J* = 8.1 Hz, 2H), 1.19 (d, *J* = 6.9 Hz, 6H).

#### 4.1.17. Synthesis of 4-(((1-(2,4-Dihydroxy-5-isopropylbenzoyl)indolin-5-yl)amino)methyl)-*N*-hydroxybenzamide (**8**)

The intermediate **S-10** was subjected to lithium hydroxide assisted hydrolysis followed by carbodiimide assited amidation with NH_2_OBn using a methodology similar to that employed for the synthesis of preceeding compounds. The resulting intermediate (0.250 mg, 3.41 mmol) was dissolved in CH_2_Cl_2_ (30 mL) and BCl_3_ (1M in CH_2_Cl_2_, 2 mL) was added at 0 °C. The reaction mixture was stirred at room temperature for 45 min. The precipitates were collected, washed with H_2_O and dried under vacuum to give compound **8** in 95% yield. mp: 230–231 °C. ^1^HNMR (300 MHz, CD_3_OD): 7.65–7.68 (m, 3H), 7.29 (d, *J* = 8.1 Hz,2H), 7.14 (s, 1H), 6.59 (s, 1H), 6.45 (d, *J* = 7.8 Hz, 1 H), 6.38 (s, 1H), 4.71 (s, 2H), 4.10 (t, *J* = 8.1 Hz, 2H), 3.23 (m, 1H), 3.06 (t, *J* = 8.1 Hz, 2H), 1.21 (d, *J* = 6.9 Hz, 6H). ^13^CNMR (75 MHz, DMSO-d_6_): 172.55, 170.84, 167.60, 167.36, 164.59, 160.39, 157.18, 153.74, 134.47, 132.08, 129.11, 128.79, 128.25, 127.43, 126.06, 115.44, 102.96, 60.26, 49.43, 46.67, 26.35, 23.13, 21.52. HRMS (ESI) for C_26_H_27_N_3_O_5_ [M-H]^−^: calcd, 460.1872; 460.1867.

### 4.2. Cell Culture

Human retinal pigment epithelial cells (ARPE-19) were purchased from Bioresource Collection and Research Center (Hsinchu, Taiwan). Dulbecco’s modified Eagle’s medium (Gibco Laboratories, Grand Island, NY, USA) with 10% *w*/*v* fetal bovine serum (Gibco Laboratories, NY, USA), 100 units/mL penicillin (Biological Industries, Beit Haemek, Israel), 0.1 mg/mL streptomycin (Biological Industries, Beit Haemek, Israel), and 2.5 × 10^−4^ mg/mL amphotericin (Biological Industries, Beit Haemek, Israel) was used to prepare cell media. The cells were incubated in 10 cm dishes at 37 °C and 5% CO_2_.

### 4.3. MTT Assay

ARPE-19 cells were seeded at a density of 2 × 10^4^ in a 48-well plate. After 24 h, cells were treated with varying concentrations of G570 or different intensities of blue light (BL). Following 24 h, 0.4 mg/mL methylthiazolyldiphenyl-tetrazolium bromide (MTT) (Sigma-Aldrich, Dublin, Ireland) was added to the cells and incubated for 2 h at 37 °C. After this MTT was removed, formazan crystals were dissolved in DMSO (Sigma-Aldrich, Dublin, Ireland). The readings were measured at an absorption of 570 nm using Sunrise™ (TECAN, Zurich, Switzerland).

### 4.4. Cell Migration Assay

ARPE-19 cells were seeded at a density of 3 × 10^5^ in 3.5 cm dishes with a silicone insert. After 48 h, the silicone insert was removed, and the cells were exposed to 150 lux blue light and treated with 0.5 µM G570. After the following 24 and 48 h, ARPE-19 cells were observed and recorded by an Olympus IX70 inverted tissue culture microscope (Olympus, Tokyo, Japan) and an SGHD-3.6C charge-coupled device (CCD) imaging system (SAGE Vision, New Taipei City, Taiwan), respectively. The occupied area of ARPE-19 cells was evaluated by ImageJ software (National Institutes of Health, Bethesda, MD, USA).

### 4.5. HeLa Nuclear Extract HDAC Activity Assay

An HDAC Fluorescent Activity Assay Kit (BioVision, Milpitas, CA, USA) was used to detect HDAC activity in the HeLa nuclear extract according to the manufacturer’s instructions. Briefly, the HDAC fluorometric substrate and assay buffer were added to HeLa nuclear extracts in a 96-well format and incubated at 37 °C for 30 min. The eaction was stopped by adding lysine developer to the cells which were incubated for another 30 min at 37 °C. A fluorescence plate reader with excitation at 355 nm and emission at 460 nm was used to quantify HDAC activity.

### 4.6. Heat Shock Protein (Hsp) 90α Activity Assay

An Hsp90α activity assay kit (BPS Bioscience, San Diego, CA, USA) was used to determine the compound inhibitory effect of Hsp90α activity according to the manufacturer’s instructions. Briefly, the reactions were conducted at room temperature for 3 h in a 100 μL mixture containing FITC-labeled geldanamycin, Hsp90α enzyme, and test compounds in assay buffer. A fluorescence polarization plate reader with excitation at 475–495 nm and emission at 518–538 nm was used to quantify Hsp90α activity.

### 4.7. HDAC Enzyme Inhibition Assays

Enzyme inhibition assays were performed by the Reaction Biology Corporation, Malvern, PA. The substrate for HDAC-1, -3, and -6 is a fluorogenic peptide derived from p53 residues 379–382 [RHKK(Ac)]. Compounds were dissolved in DMSO and tested in 10-dose IC_50_ mode with 3-fold serial dilution starting at 10 μM. Trichostatin A (TSA) was used as the reference.

### 4.8. Western Blot Assay

ARPE-19 cells were seeded at a density of 4 × 10^5^ in 6 cm dishes. After 24 h, cells were treated with 0.5 µM G570 and 150 lux blue light. After 24 h, cells were harvested in RIPA lysis buffer (50 mM Tris-HCl, 150 mM NaCl, 1 mM EGTA, and 1% *w*/*v* Nonidet P-40) and placed on ice. Collected cells were centrifuged at 14,000 g for 10 min. Total protein was collected, and the concentration was measured using Bradford reagent (Bio-Rad Laboratories, Hercules, CA, USA). Total proteins were isolated by SDS-PAGE electrophoresis (Bio-Rad Laboratories, CA, USA) and transferred onto PVDF membranes (Millipore Sigma, Burlington, MA, USA). After blocking with 5% *w*/*v* BSA (Sigma-Aldrich, Dublin, Ireland) for 40 min, the membranes were incubated with primary antibodies against 1:500 VEGFA-189 (Novus Biologicals, Centennial, CO, USA), 1:500 VEGFR2 (Novus Biologicals, CO, USA), 1:1000 ERK (Cell Signaling, Danvers, MA, USA), 1:1000 FAK (Abcam, Cambridge, UK), 1:1000 p38 (Cell Signaling, MA, USA), 1:2000 Akt (Cell Signaling, MA, USA), and 1:5000 eNOS (BD, Franklin Lakes, NJ, USA). Then, the membranes were washed twice with TBST and probed with 1:5000 goat anti-rabbit IgG (HRP) or 1:5000 goat anti-mouse IgG (HRP) (GeneTex, Irvine, CA, USA). The expression of these proteins was detected with HRP substrate luminol reagent by a BioSpectrum^®^ 500 Imaging System (UVP, Upland, CA, USA).

### 4.9. Animals and Treatment

Male C57BL/6 mice (6–8 weeks old) were obtained from the National Laboratory Animal Center and housed aseptically in Taipei Medical University animal facilities. All animal procedures were approved by the Institutional Animal Care and Use Committee (IACUC, LAC-2018-0166) of Taipei Medical University and conducted according to guidelines of the Association for Research in Vision Ophthalmology (ARVO) statement for the use of animals in ophthalmic and vision research. The animals were randomly divided into experimental groups and subjected to the corresponding treatment.

### 4.10. Laser-Induced Choroidal Neovascularization (CNV) Model and Injection

C57BL/6 mice were given general anesthesia using isoflurane (PBF, Taipei, Taiwan) and mydriatic eyes were treated with 1% *w*/*v* tropicamide (Alcon, Geneva, Switzerland) before experiments. To analyze the efficacy of G570, C57BL/6 mice were divided into three groups and treated with vehicle control, *n* > 3 eyes (1% DMSO in saline), G570, *n* > 3 eyes (20 mg/kg/day, i.p.), and Eylea (Bayer, Germany), *n* > 3 eyes (40 μg/1 μL per eye, i.v.t.). All mice were treated with the drugs, as represented in Figure 6B. The use of positive control (Eylea) was according to Miciulaitiene et al., 2018 [41], and the dose-finding in our lab. Our design for Eylea for one-dose/one-time treatment due to its good efficacy and invasive effect. G570 has administrated four doses at days 0, 1, 3, 5. FFA imagine analysis was collected on day 7, 0, 7, 14, and day 21. Similar to Eylea, Intraperitoneally injection also is an invasive treatment for mice. We choose every two days to give the i.p. administration. 

Laser photocoagulation was performed by an Imagine Guide Laser system (Phoenix Research Laboratories, Tempe, AZ, USA). Briefly, four thermal burns were induced in each eye around the optic nerve using a slit lamp delivery system at day 0.

### 4.11. Fundus Angiography Analysis

Prior to analysis, the pupil of the right eye of each mouse was dilated with 0.125% atropine sulfate. The whiskers were trimmed with scissors. The position and angle were adjusted to favor the investigation of various parts of the fundus. The eyes were covered with 2% methodical gel (OmniVision, SA, Neuhausen, Switzerland), and fundus images and fluorescein angiography images were captured using Phoenix MICRON™ III (Phoenix Research Laboratories, Tempe, AZ, USA). For fluorescein angiography, 10% sodium fluorescein was injected into the mice intraperitoneally. Images were captured after 30 s of injection. Healthy profiles of C57BL/6 mice were recorded in FFA figures 7 days before laser CNV induction. After laser injury, G570 was intraperitoneally administered (20 mg/kg per day) on days 0, 1, 3, and 5. The Eylea group, which served as the positive control, was treated by intravitreal injection (40 μg/1 μL per eye) on day 0. FFA figures were also recorded on days 7, 14, and 21. All figures were transferred to black–white images, and the leakage area of fluorescein was evaluated by ImageJ software. The G570 solution was prepared by dissolving 2 mg G570 in 50 µL DMSO (Sigma, St. Louis, MO, USA). Then, 50 µL Cremophor^®^ A6 (Wei Ming Pharmaceutical MFG, R.O.C., Taipei, Taiwan) and 900 µL saline were added one after the other. Data were quantified using Image-Pro software (Media Cybernetics, Bethesda, MD, USA).

### 4.12. Statistical Analysis

All experiments were conducted in triplicate. Data are presented as the mean ± S. D. All in vitro and in vivo results were analyzed by one-way analysis of variance (ANOVA) followed by Scheffe post hoc test to calculate the *p*-value for each group. All statistical analyses were performed using IBM SPSS Statistics 19 software (IBM, Armonk, NY, USA). Differences between each condition were considered to be significant when * *p* ≤ 0.05, ** *p* ≤ 0.01, and *** *p* ≤ 0.001; **^#^** *p* ≤ 0.05, **^##^** *p* ≤ 0.01, and **^###^** *p* ≤ 0.001.

## Figures and Tables

**Table 1:** Inhibition of HeLa nuclear extract HDAC activity and HSP90 chaperone protein by compounds **1**–**8**.

Compounds	HSP90 IC_50_ (nM)	HeLa HDAC IC_50_ (nM)
**1**	49.3 ± 1.6	>1000
**2**	55.9 ± 3.2	862.9 ± 55.3
**3**	N.D.	N.D. ^a^
**4**	40.2 ± 5.2	86.9 ± 14.4
**5**	46.3 ± 7.5	61.2 ± 2.0
**6**	52.1 ± 2.9	174.4 ± 5.9
**7**	58.6 ± 2.2	104.7 ± 14.8
**8**	85.7 ± 8.6	>1000
**SAHA**	>1000	99.4 ± 17.7
**BIIB021**	65.7 ± 1.6	>1000

The enzyme inhibitory potential of the synthesized compounds was evaluated against HeLa nuclear extract as the HDAC source and HSP90. Careful observation of Table 1 indicates that all the compounds were able to inhibit the HSP90 chaperone with IC_50_ values ranging from 49 to 85 nM; however, Hela HDAC did not exhibit sensitivity to compound **1** bearing a short linker (long-chain alkyl linker bearing compound, *n* = 2) and the benzyl linker containing compound (compound **8**). Overall, compound **4** and compound **5** were identified as the most potent inhibitors of HeLa HDAC as well as HSP90. ^a^ Not detectable.

**Table 2:** HDAC isoform inhibitory effects of compounds **1**–**8**.

Compds	HDAC1 IC_50_ (µM)	HDAC3 IC_50_ (µM)	HDAC6 IC_50_ (nM)	HDAC8 IC_50_ (µM)	Selectivity Ratio		
					HDAC 1/6	HDAC 3/6	HDAC 8/6
**1**	-	-	>10,000	-	-	-	-
**2**	2.99	0.544	9.75	1.96	306	55	201
**3**	4.45	2.65	26.1	2.94	170	101	112
**4**	0.44	0.34	2.13	1.55	206	159	727
**5 (MPT0G570)**	0.13	0.16	1.15	0.284	113	139	246
**6**	1.95	1.81	9.25	1.22	210	195	131
**7**	2.03	1.89	17.3	1.67	117	109	96
**8**	-	-	74.4	1.55	-	-	20
**Trichostatin A**	0.0069	0.0104	2.60	1.02	2.65	4	392

The compounds were further evaluated for HDAC isoform inhibitory effects against HDAC 1, 3, 6, and 8 (Table 2). Trichostatin A was employed as the standard. The results of the assay demonstrated a striking selective inhibitory profile of the compounds towards the HDAC6 isoform. Several compounds of the series were able to inhibit the HDAC6 isoform at the single-digit nanomolar concentration (compound **2**, **4**, **5**, **6**). Amongst them, compound **5** was identified as the most potent inhibitor with an IC_50_ value of 1.15 nM and a selectivity ratio of **113** or HDAC1/HDAC6, **139** for HDAC3/HDAC6, and **246** for HDAC8/HDAC6. Other than the HDAC6 isoform, most of the compounds also exhibited inhibitory potential towards HDAC 1, 3, and 8 at micromolar or submicromolar concentrations.

## Data Availability

Data is contained within the article.

## Supplementary Materials

The following are available online, HPLC purity data SI-2; 1H NMR Spectra for compounds **1**–**8**.
