# Supplementary material for: The HDAC/HSP90 Inhibitor G570 Attenuated Blue Light-Induced Cell Migration in RPE Cells and Neovascularization in Mice through Decreased VEGF Production
Source: Molecules. 2021 Jul 19;26(14):4359. doi: 10.3390/molecules26144359 (PMC8305912; doi:10.3390/molecules26144359)
Supplement: Supplementary file 1 [file molecules-26-04359-s001.zip › molecules-1194018-supplementary.pdf]

molecules

Article

The HDAC/HSP90 Inhibitor G570 Attenuated Blue Light-Induced Cell Migration in RPE Cells and Neovascularization in Mice through Decreased VEGF Production

Tai-Ju Hsu [1,†], Kunal Nepali [1,†], Chi-Hao Tsai [1,2], Zuha Imtiyaz [1], Fan-Li Lin [3], George Hsiao [3], Mei-Jung Lai [4,5,*], and Yu-Wen Cheng [1,4,*]

[1] School of Pharmacy, College of Pharmacy, Taipei Medical University, Taipei 100301, Taiwan; nick010519@tmu.edu.tw (T.-J.H.); nepali@tmu.edu.tw (K.N.); d01447001@ntu.edu.tw (C.-H.T.); zuhaimtiyaz@tmu.edu.tw (Z.I.)
[2] Department of Ophthalmology, School of Medicine, the University of North Carolina at Chapel Hill, Chapel Hill 27599, USA.
[3] Department of Pharmacology, School of Medicine, College of Medicine, Taipei Medical University, Taipei 100301, Taiwan; fllin@tmu.edu.tw (F.-L.L.); geohsiao@tmu.edu.tw (G.H.)
[4] Ph.D. Program in Drug Discovery and Development Industry, College of Pharmacy, Taipei Medical University, Taipei 100301, Taiwan
[5] Biomedical Commercialization Center, Taipei Medical University, Taipei 100301, Taiwan
* Correspondence: mjl@tmu.edu.tw (M.-J.L.); ywcheng@tmu.edu.tw (Y.-W.C.); Tel.: +886-2-27361661 (ext. 7686) (M.-J.L.); +886-2-27361661 (ext. 6123) (Y.-W.C.); Fax: +886-2-27374622 (Y.-W.C.);
† These authors contributed equally to this work.

Contents:

HPLC purity data……………………….……………………………………….SI-2

^{1}H NMR Spectra for compounds 1-8………………………………………… .SI-3-10

HPLC Purity Data of compounds 1-8

HPLC purity determination:

HPLC purity was determined using an Hitachi 2000 series HPLC system using C-18 column (Agilent ZORBAX Eclipse XDB-C18 5 µm. 4.6 mm × 150 mm). Elution conditions: Mobile phase A-Acetonitrile; Mobile phase B-Water containing 0.1% formic acid + 10 mmol NH_4OAc. The flow-rate was 0.5 mL/min and the injection volume was 5 µl. The system operated at 25 °C. Peaks were detected at 254 nm.

Compound	Retention time (min)	Purity (%)
**1**	3.104	96.281
**2**	3.108	99.189
**3**	3.173	99.431
**4**	16.54	97.618

**5**	17.67	96.475
**6**	18.84	98.886
**7**	18.75	98.959
**8**	16.93	97.929

¹H Spectra for compound 1.

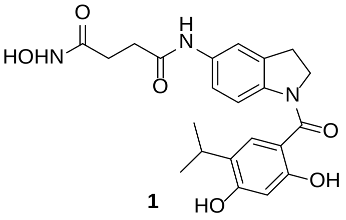

1

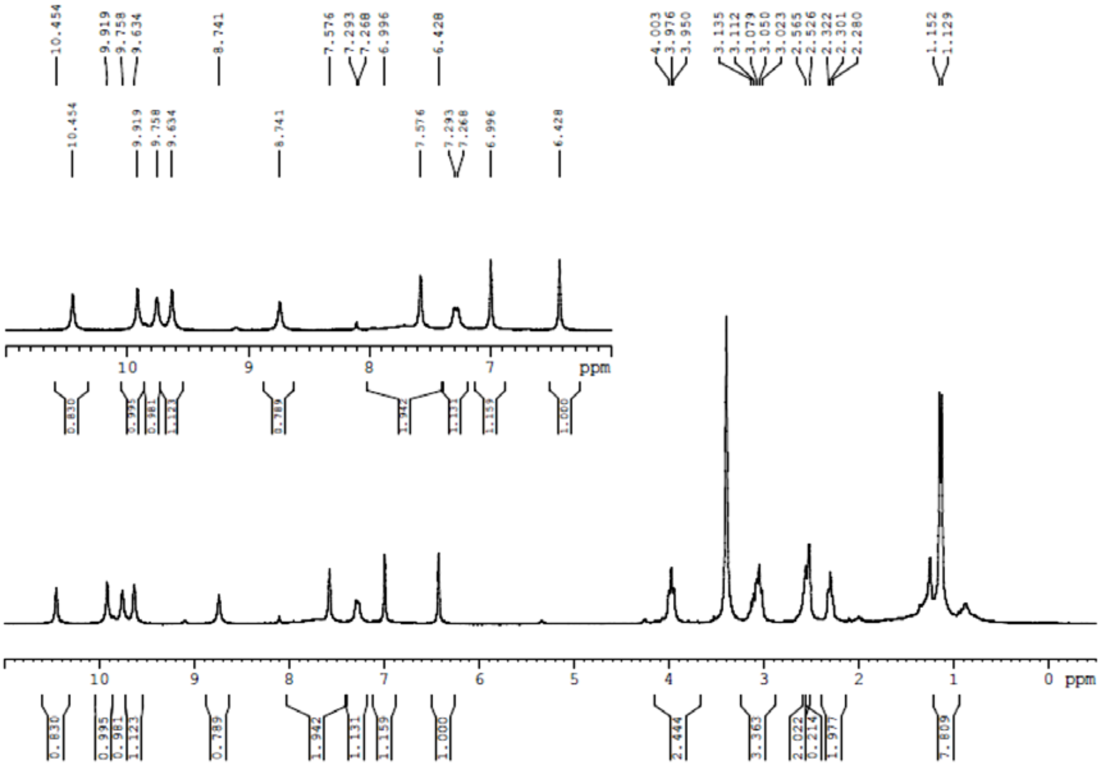

¹H Spectra for compound 2.

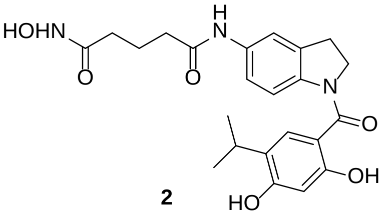

2

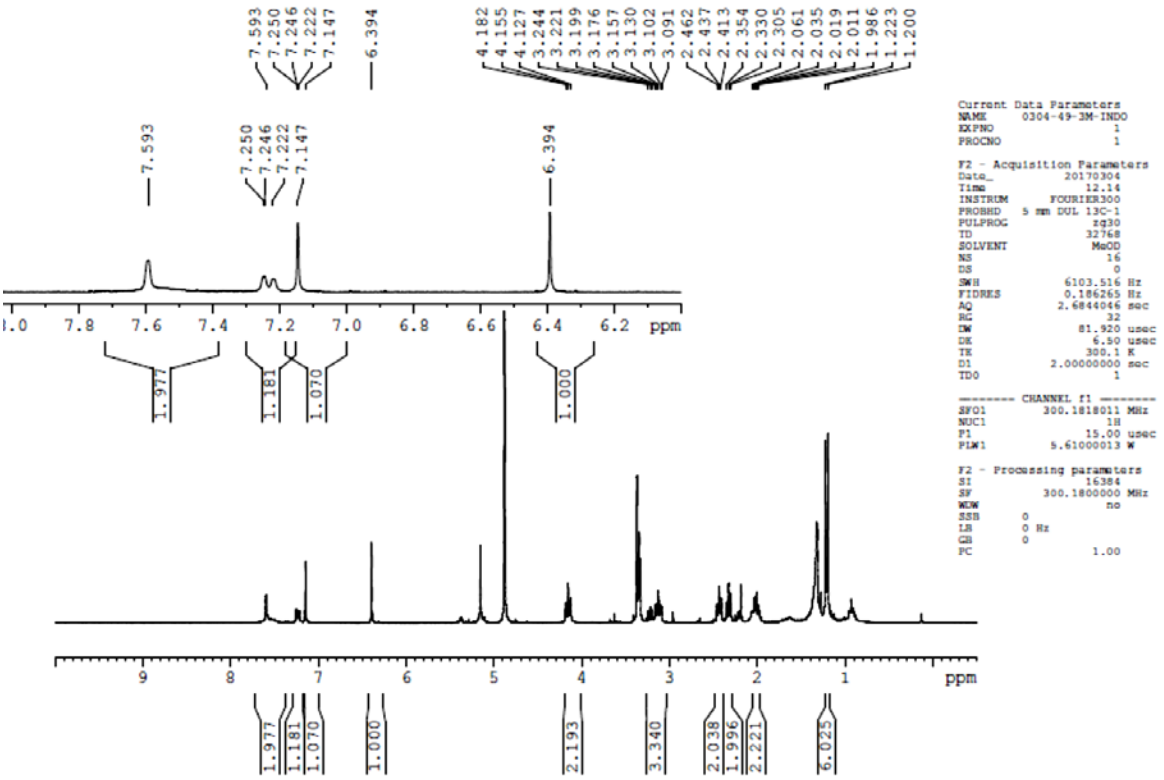

^{1}H Spectra for compound 3.

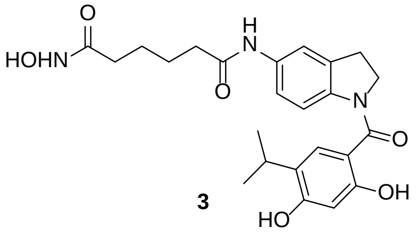

3

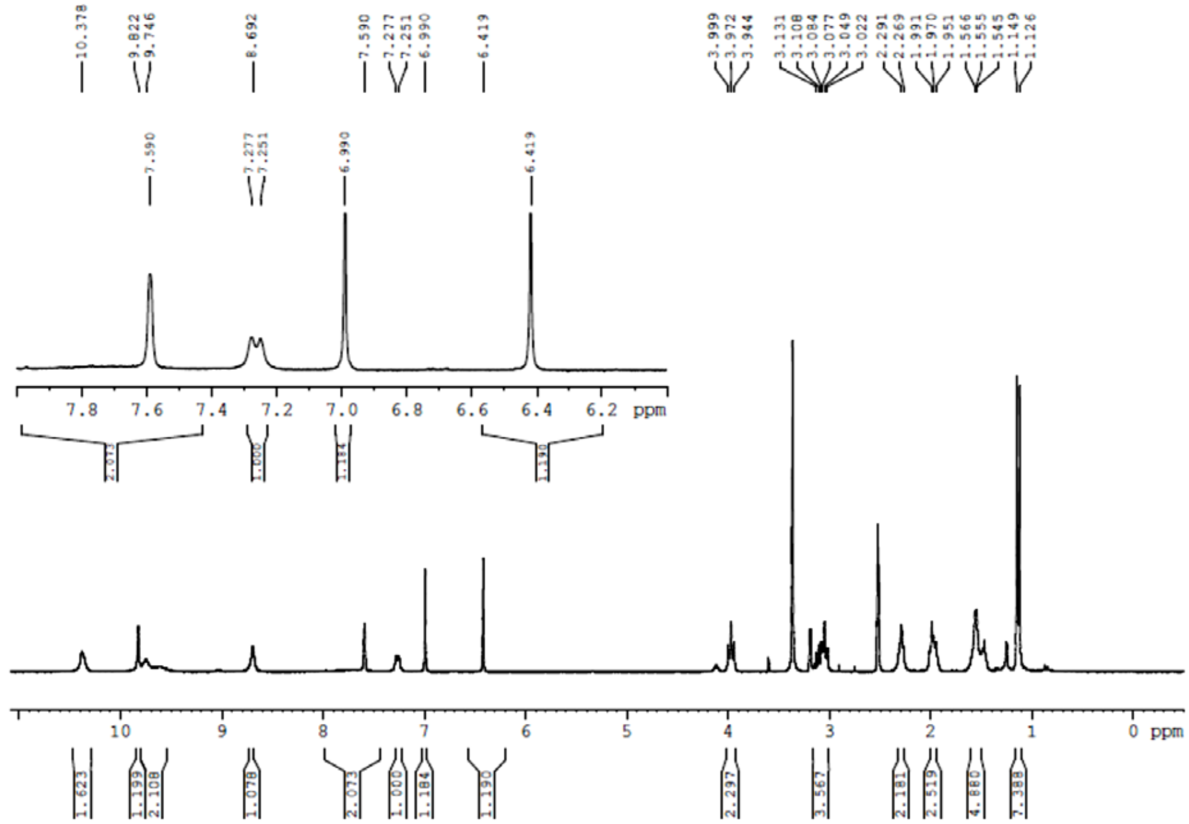

^{1}H NMR of compound 4.

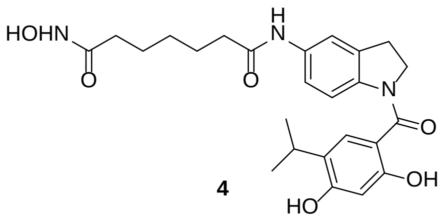

4

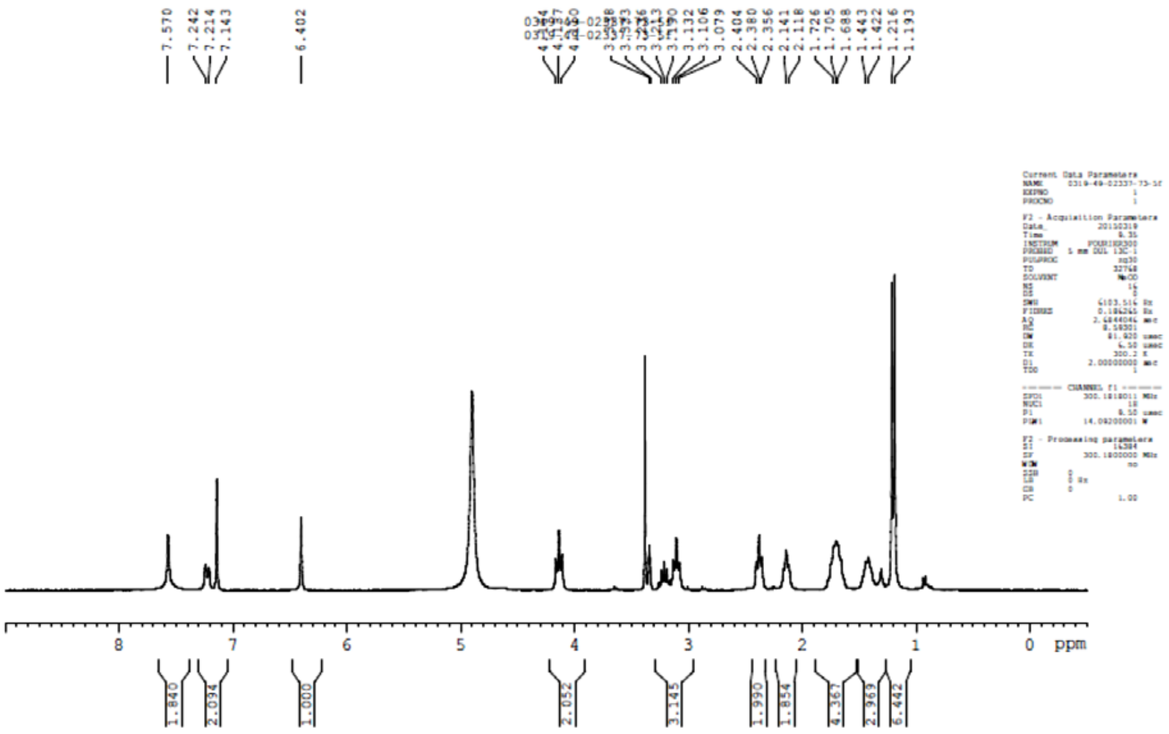

^{1}H NMR of compound 5.

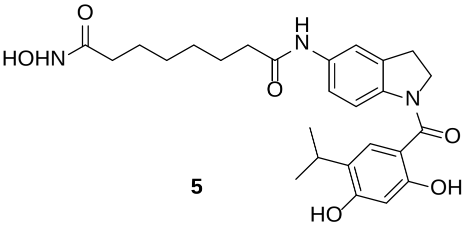

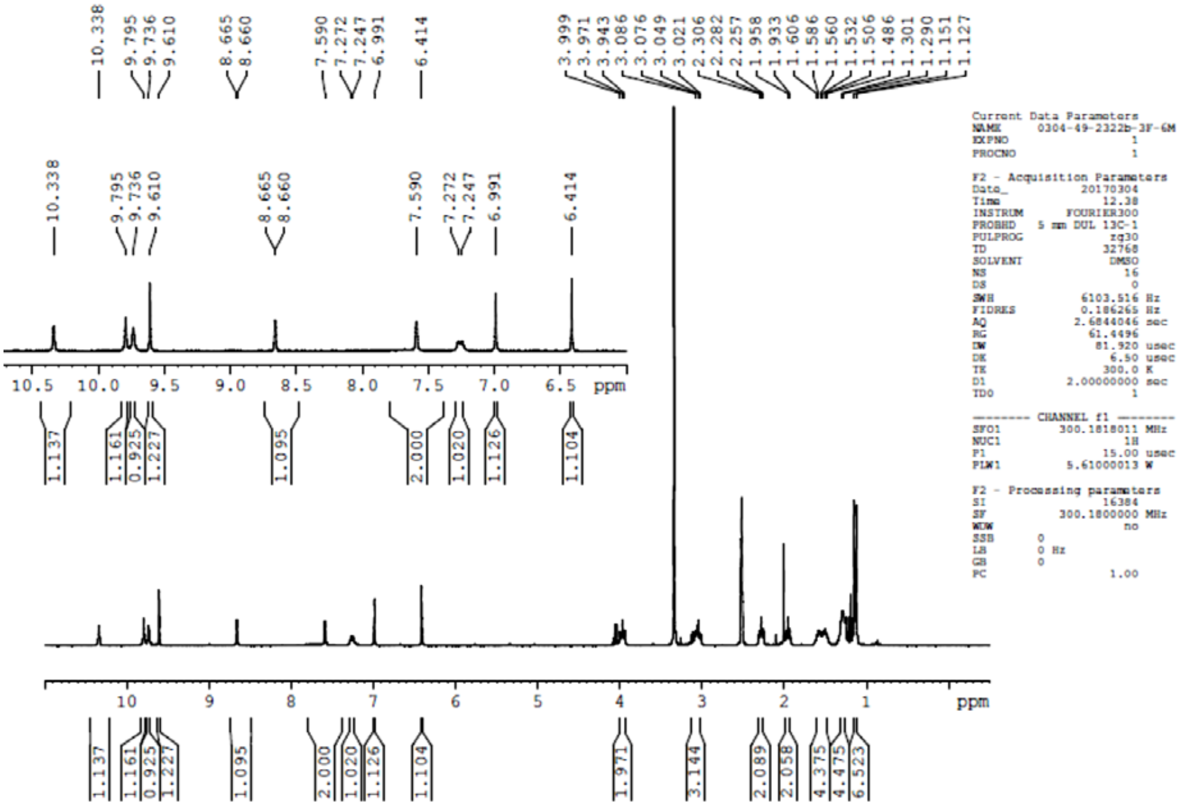

^{1}H NMR of compound 6.

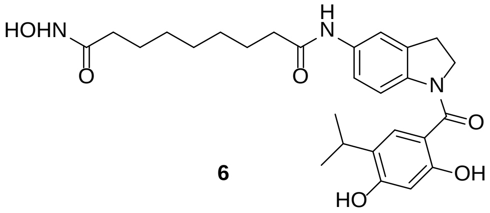

6

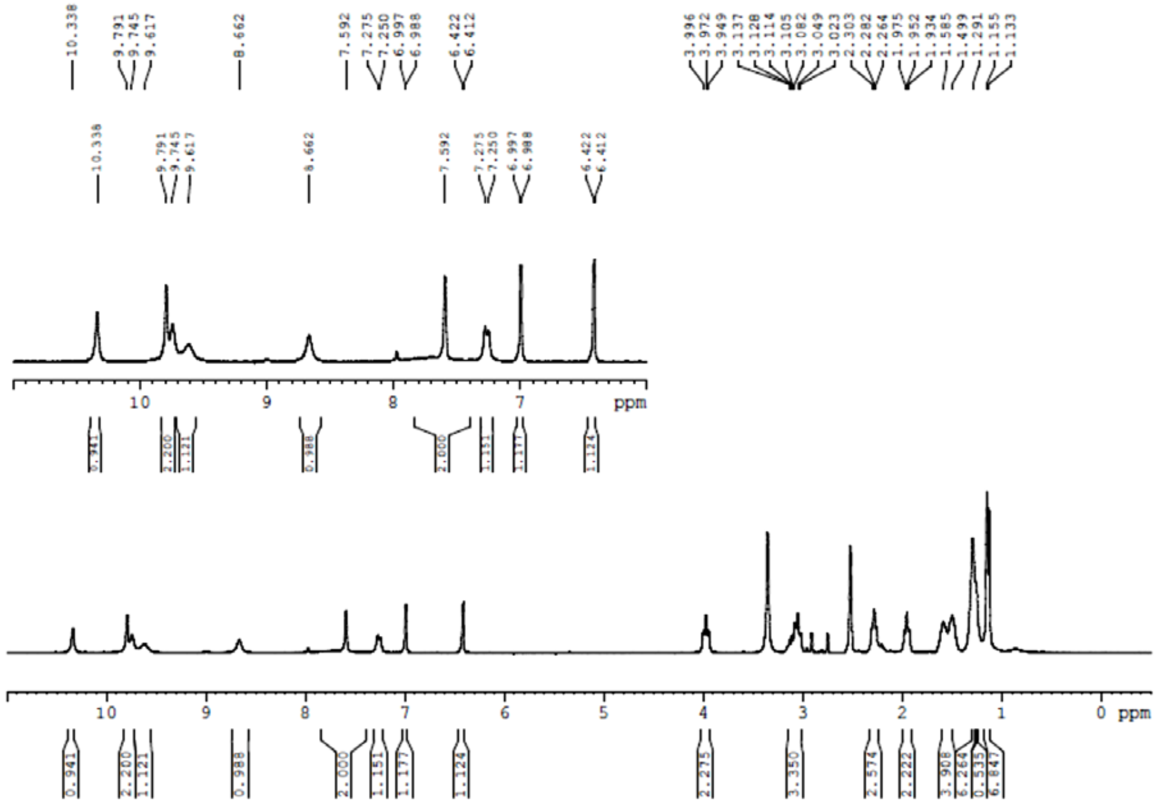

^{1}H NMR of compound 7.

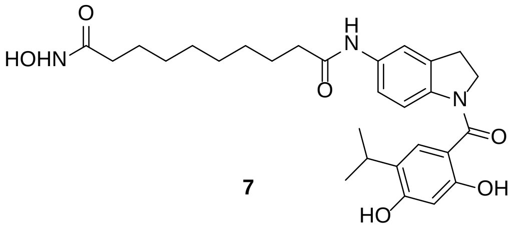

7

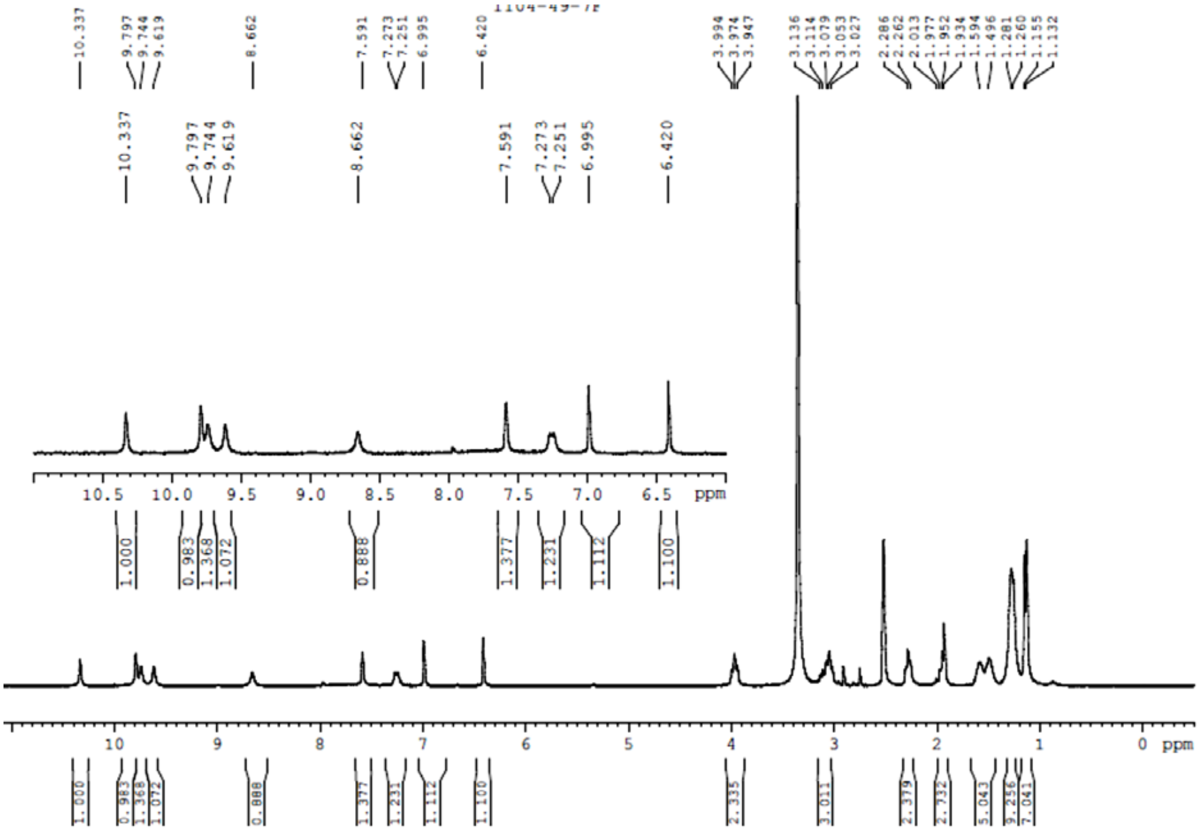

^{1}H NMR of compound 8.

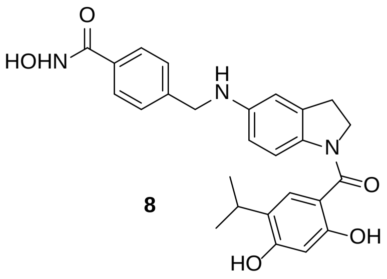

8

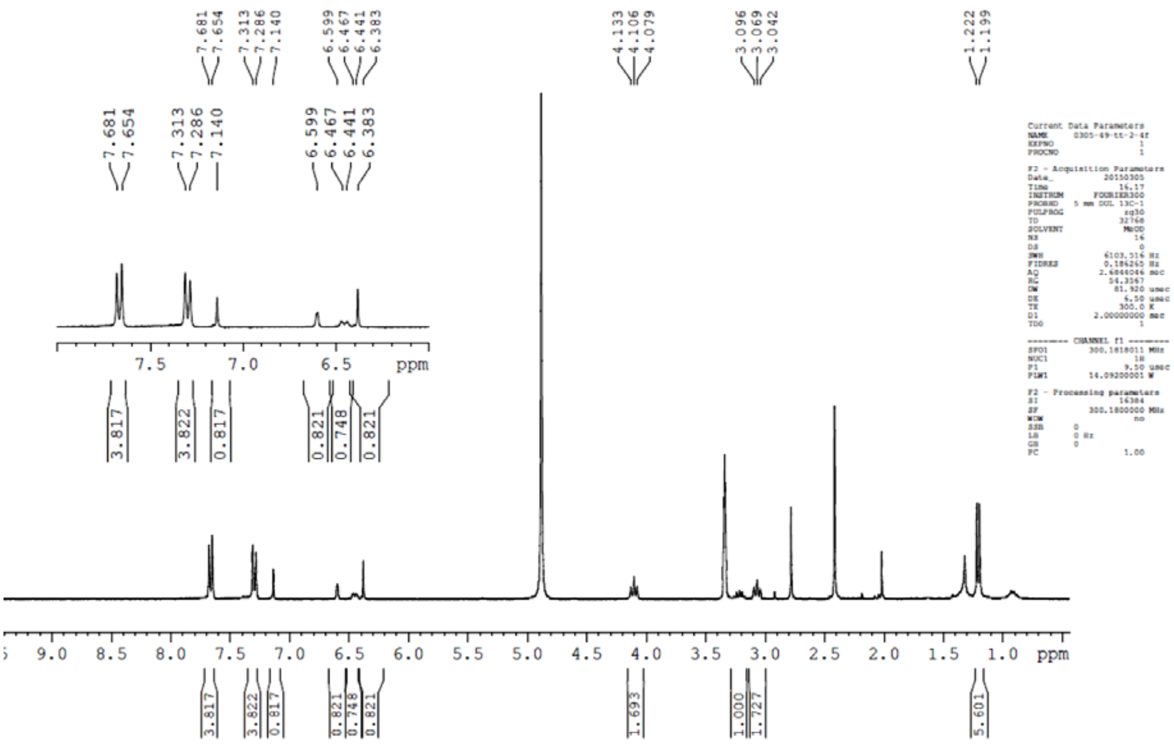
7.681
7.654
7.313
7.286
7.140
6.599
6.467
6.441
6.383
4.133
4.106
4.079
3.096
3.069
3.042
1.222
1.199
3.817
3.822
0.817
0.821
0.748
0.821
1.693
1.000
1.727
5.601
ppm
